# Author Correction: Tunable encapsulation of sessile droplets with solid and liquid shells

**DOI:** 10.1038/s41467-024-47609-6

**Published:** 2024-04-12

**Authors:** Rutvik Lathia, Satchit Nagpal, Chandantaru Dey Modak, Satyarthi Mishra, Deepak Sharma, Bheema Sankar Reddy, Pavan Nukala, Ramray Bhat, Prosenjit Sen

**Affiliations:** 1 Centre for Nano Science and Engineering, Indian Institute of Science, Bangalore, 560012 India; 2 Department of Developmental Biology and Genetics, Indian Institute of Science, Bangalore, 560012 India; 3 Department of BioSystems Science and Engineering, Indian Institute of Science, Bangalore, 560012 India

**Keywords:** Chemical engineering, Physical chemistry

Correction to: *Nature Communications* 10.1038/s41467-023-41977-1, published online 13 October 2023

The original version of this Article contained an error in the 12th sentence of the first paragraph of the ‘Results and Discussion’ section, which incorrectly read ‘The adhesive force on a particle scales as *γ*_ow_*R*_p_(1 + cos *θ*_ow_) ≈ 10^−8^ N, where *θ*_ow_ is the contact angle of the oil-water interface with the particle.’ The correct version replaces this sentence with ‘The adhesive force on a particle scales as *πγ*_ow_*R*_p_cos^2^(*θ*_ow_/2) ≈ 10^−8^ N, where *θ*_ow_ is the contact angle of the oil-water interface with the particle (here, *γ*_ow_ = 42 mN m^−1^, *R*_p_ = 17.5 µm, and *θ*_ow_ = 170°).’. This has been corrected in both the PDF and HTML versions of the Article.

