# Supplementary material for: Tunable encapsulation of sessile droplets with solid and liquid shells
Source: Nat Commun. 2023 Oct 13;14:6445. doi: 10.1038/s41467-023-41977-1 (PMC10575970; doi:10.1038/s41467-023-41977-1)
Supplement: Supplementary file 1 — Supplementary Information File [file 41467_2023_41977_MOESM1_ESM.pdf]

Supplementary Information for

Tunable Encapsulation of Sessile Droplets with Solid and Liquid Shells

Rutvik Lathia[1,+], Satchit Nagpal[1,+], Chandantaru Dey Modak[1], Satyarthi Mishra[1], Deepak Sharma[1], Bheema Sankar Reddy[1], Pavan Nukala[1], Ramray Bhat[2,3], and Prosenjit Sen[1,3,*]

[1] Centre for Nano Science and Engineering, Indian Institute of Science, Bangalore, India, 560012

[2] Department of Developmental Biology and Genetics, Indian Institute of Science, Bangalore, India, 560012

[3] Department of BioSystems Science and Engineering, Indian Institute of Science, Bangalore, India, 560012

[+] **These authors contributed equally.**

***Corresponding Author's Email:** prosenjits@iisc.ac.in

Supplementary note 1. Comparison with composite LM configuration

	Composite LM	LMOI (This work)
**Uniform shell**	×	✓
**Tunability**	×	✓
**Lifetime**	< 3 h	>12 days
**High-Temperature stability**	×	✓
**Hanging configuration**	×	✓
**Liquid sampling without disruption**	×	✓
**Single crystal growth**	×	✓
**Cell growth**	×	✓
**Particle removal on-demand**	×	✓
**High mechanical stability**	✓	✓
**Transparency**	× (because of clumps)	✓
**Solid capsule formation**	×	✓
**Stimuli-responsiveness**	×	✓

Supplementary Table 1: Comparison of composite LM with LMOI. × and ✓ symbol represent the not possible and possible scenarios with the listed technique.

In composite LM, the oil layer is spin-coated on a surface, and then a water droplet is made to slide over it.[1] The sliding water droplet gets covered with a thin layer of oil. The oil-coated droplet is detached from the surface and then rolled over a particle bed. This makes a composite LM structure with a combination of oil and particles. This method is not suitable for making uniform shells which are tunable over a wide range of thickness. In comparison, the method reported in this manuscript can achieve a wider range of oil thickness starting from 5 μm to 200 μm.

Supplementary note 2. Problems of thickness tunability in composite LM

It can be conceived that a thicker encapsulation should be possible by using larger oil volumes for pre-coating the water droplet. However, this approach is not feasible as the oil coating on the droplet becomes non-uniform. Supplementary Fig. 1 shows the effect of using higher oil volumes for coating a 10 μL water droplet. The balance of surface and gravitational forces leads to a non-uniform oil layer thickness over the droplet (see Supplementary Fig. 1).[2–5] Further, during the sliding and detachment of the droplet from the surface, viscosity and density differences will also contribute to the non-uniformity in the oil layer thickness. The non-uniformity in oil layer thickness results in a variable particle coating.

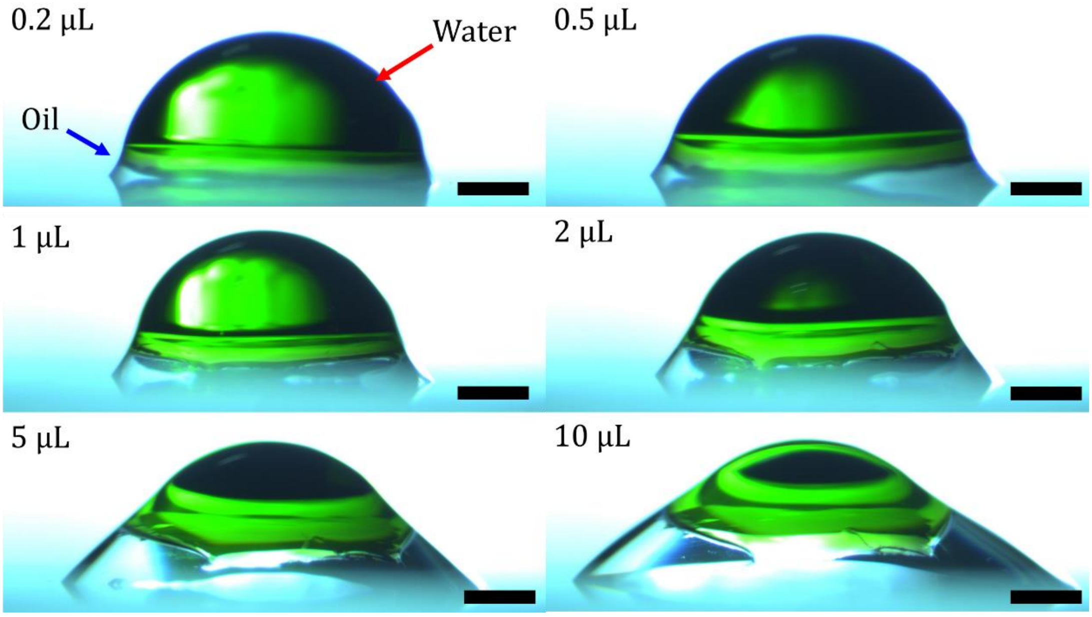

Supplementary Fig. 1: Droplet covered with oil. The sessile water droplet (10 μL) covered with different amounts of silicone oil (20 cP) on a Teflon-coated glass. Scale bar = 0.5 mm.

Apart from the oil encapsulation in composite LM, it is also very difficult to get a uniform coating of oil over a droplet with other methods. Such methods include concentric nozzle/needle, where the core liquid can be ejected from the central needle, and shell material can be injected through the outer needle. However, the uniformity of the shell is still very hard

to achieve and limited to some special liquids only. Even a slight variation in the density between core and shell liquid can significantly impact the mechanical stability of such droplets.[6,7] When a compound drop, consisting of a core phase and a surrounding shell phase, descends through the air, the core phase tends to accelerate downward if it has a higher density compared to the shell phase. Conversely, if the core phase is less dense, it tends to move upward. Thus, the above problem forces the needle-based systems to have a density match between core and shell liquids and thereby limiting the choices of liquids.[8]

In addition to the challenges posed by the non-uniformity in coating oil before particles, the method described above also encounters issues with particle clumping, which introduces additional non-uniformity in the coating process. When oil-coated droplets impact the particle bed, the oil tends to seep through the bed, resulting in the formation of clumps or agglomerations at the interface where the oil thickness exceeds the size of the particles. These clumps significantly contribute to the non-uniformity of the coating, as depicted in Supplementary Fig. 2. The timescale of wetting in porous media is governed by the Washburn equation if $\eta \gg \rho^{\frac{3}{2}} g R^{\frac{5}{2}} \gamma^{-\frac{1}{2}}$.[9] For $\rho \approx 1000\ \mathrm{kg\ m^{-3}}$, $R \approx 35\ \mu\mathrm{m}$ and $\gamma \approx 20\ \mathrm{mN\ m^{-1}}$, $\rho^{\frac{3}{2}} g R^{\frac{5}{2}} \gamma^{-\frac{1}{2}} \approx 10^{-5}\ \mathrm{Pa\ s}$ which is around two to four orders of magnitude lower than the viscosity used ($\eta \approx 10^{-3} - 10^{-1}$ Pa s) in the present paper; thus, the Washburn equation is valid in our case as well.

According to the Washburn equation, the time of wetting is given by $\tau = 4\eta l^2 \gamma^{-1} D_{\mathrm{p}}^{-1}$, where l is the wetting length (≈ two particle layers for clump formation ≈ 70 µm) and D_{p} is the diameter of the particle (35 µm). Thus, assuming clumps of particles occur as wetting takes

place over two layers, the minimum time required for clump formation is estimated as ≈ 0.28 ms. The timescale of contact for the impact of such a droplet is in the order of 10 ms, which is 2 orders of magnitude higher than the clump formation time scale. Therefore, in a very short period of time, the multiple layers of particles in the particle bed get wetted and adhere to the LM surface, which causes non-uniform clumps all around the composite LM. This also explains the reason behind having a higher oil layer thickness compared to particle size in composite LM literature.[1] Thus, particle coating has to be done before oil coating to ensure uniform and tunable coating throughout the interface.

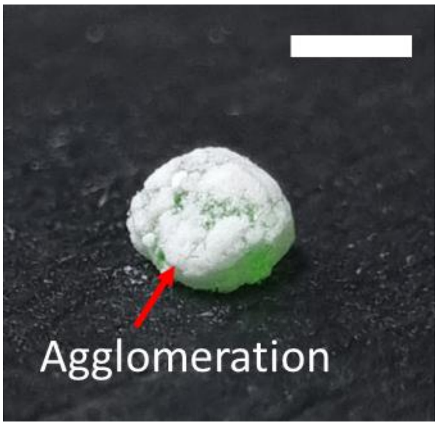

Supplementary Fig. 2: Composite LM. The composite LM prepared by the technique mentioned in Ref.[1] by using silicone oil with 35 µm particle size. Scale bar = 2 mm.

We evaluated the effect of clumping on the evaporation rate. Supplementary Fig. 3 represents the normalized mass evolution for the LMOI prepared by the technique reported in this paper and composite LM for 10 µL droplet. The composite LM was prepared by using 0.8 µL mineral oil to have nearly 35 µm thickness. The LMOI lifetime is around two orders of magnitude higher than the composite LM lifetime. This is because of the uneven coating of the composite LM as vapor escapes through the thinly coated sides, while in the case of LMOI, the uniform coating ensures no abrupt escape of vapor.

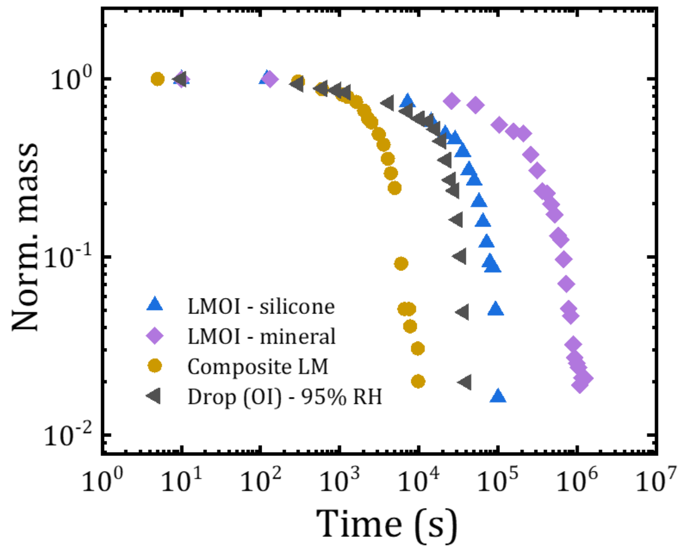

Supplementary Fig. 3: Evaporation rate comparison. The normalized mass (m/m_0) evolution for the LMOI and other techniques for 10 μL droplet. Source data are provided as a Source Data file.

Other than non-uniformity, it is very difficult to form a solid encapsulation with the composite LM. Coating wax prior to particles faces similar problems as mentioned above for coating oil first. Additionally, to prevent solidification of the wax, the high temperature has to be maintained during the whole process. This will make the setup cumbersome.

**Oil type**	**Density (kg m^{-3})**	γ_o **(mN m^{-1})**	γ_{ow} **(mN m^{-1})**
Silicone oil	920	17.4	41.97
Mineral oil	780	26.41	45.21
Neem oil	772	24.4	12.26
Almond oil	830	27.25	43.98
Mustard oil	870	31.32	23.2
Olive oil	780	28.02	42.85
Sunflower oil	850	28.46	43.11

Supplementary Table 2: Surface tension of the various liquids. The value of interfacial tension for different oils with air (γ_o) and water (γ_{ow}), calculated by the pendant drop method. The values represented in the table are mean of n = 3 independent samples.

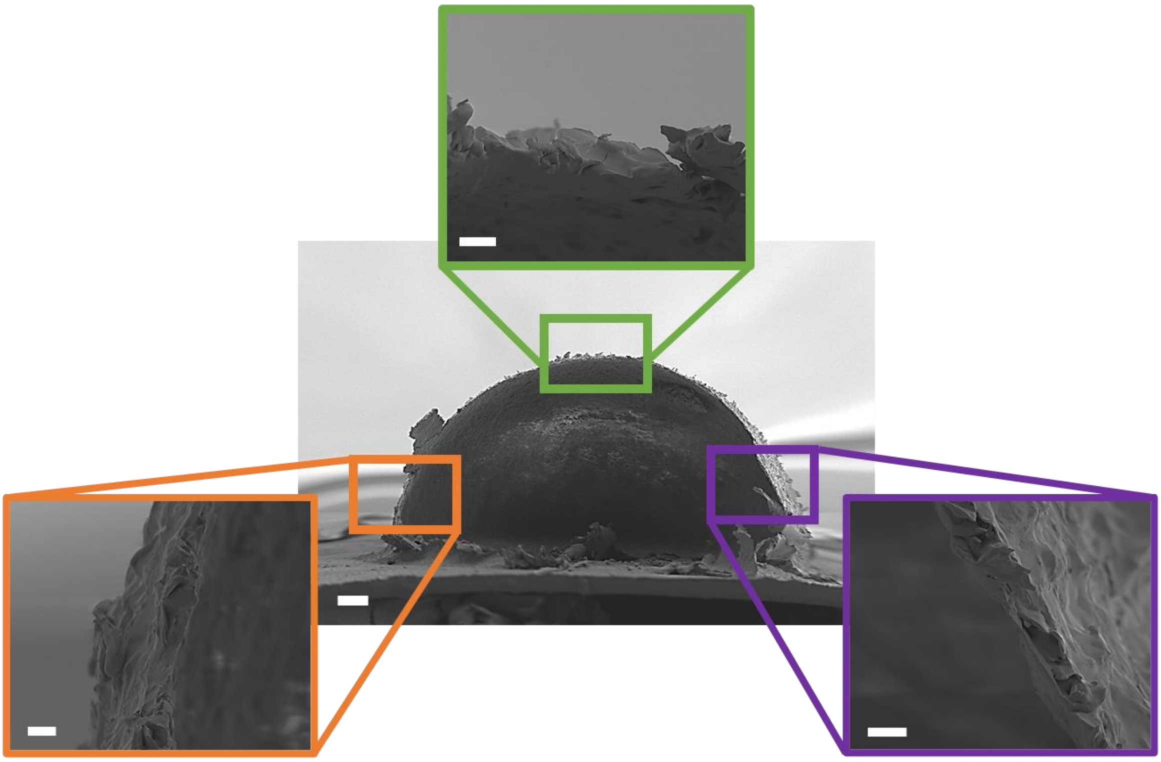

Supplementary Fig. 4: Measurement for shell thickness. SEM image of the half-cut capsule representing the various sections of the capsules, namely, bottom left, Top and Bottom right. The thickness values obtained from three independent experiments at each location. Scale bar = 200 µm for the central image. For others, Scale bar = 20 µm.

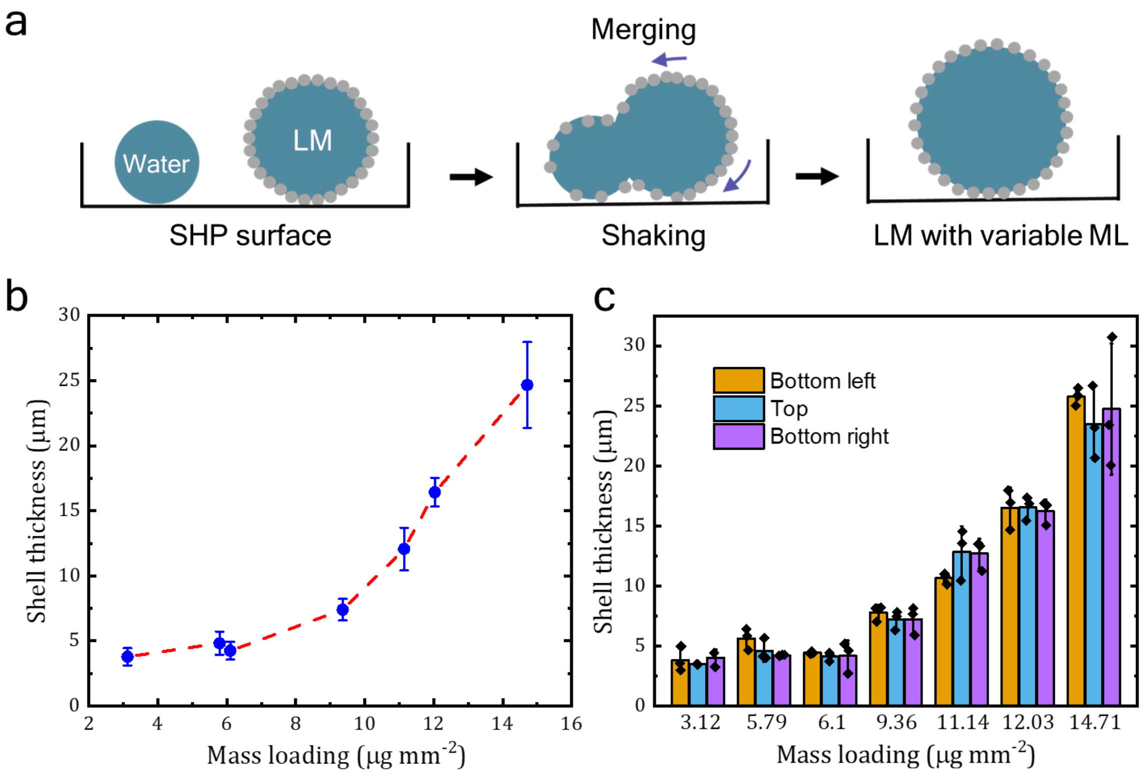

Supplementary Fig. 5: Effect of mass loading. a, The methodology to vary the mass loading (ML) of the LM is obtained by fixing the initial volume of the LM and subsequently increasing its volume to 10 µL by merging it with the bare water droplet. The whole process was carried out on a superhydrophobic surface to prevent LM from breaking. **b,** The shell thickness change with respect to the mass loading (ML) of the particles. Measurements were carried out on n = 3 independent samples, and data are presented as mean values ± SD. **c,** The shell thickness measurement for the bottom left (orange color), top (blue color), and bottom right (purple color) sections of the wax capsule for various particle mass loading with 35 µm particle size. Measurements were carried out on n = 3 independent samples, and data are presented as mean values ± SD. The black dots on the graph represent the individual data points. Source data are provided as a Source Data file.

Supplementary note 3. Particles at the interface

Particle density in LM

The particle density (mass loading) of the liquid marble has been varied by coalescing LM with bare droplets with a final volume fixed at 10 µL (Supplementary Fig. 5a). The different volumes taken for fabricating various mass-loaded LM are listed in Supplementary Table 3. Where V_{LM}, V_W and ϕ_s represent the volume of liquid marble, the volume of the water drop, and the solid surface fraction of the prepared LM. The solid fraction was determined by optical imaging using a microscope. The mass loading was determined by averaging the mass of ten liquid marbles.

V_{LM} (µL)	V_W (µL)	ML (µg mm^{-2})	ϕ_s
0	10	0	0
1.5	8.5	3.12 ± 0.15	0.64 ± 0.04
2	8	5.79 ± 0.13	0.71 ± 0.05
2.5	7.5	6.1 ± 0.39	0.73 ± 0.06
3	7	9.36 ± 0.36	0.84 ± 0.05
5	5	11.14 ± 0.35	0.89 ± 0.04
7	3	12.03 ± 0.43	0.96 ± 0.03
10	0	14.71 ± 0.40	1

Supplementary Table 3: Mass loading variation in LM. The values of volume to be taken for particular mass loading and respective solid fraction. The particle used here is 35 µm average size (obtained through sieving particles with 25 µm and 45 µm pore size). As described above, various sizes of LM and droplets collision results in different mass loading (ML).

Structure of LMOI

In the present study, we encountered challenges in directly observing the particles embedded in the cloaking layer. However, we were able to perform scanning electron microscopy (SEM) analysis on wax-coated LMOI (solid capsule) to gain insights into the distribution of polytetrafluoroethylene (PTFE) particles on the surface of the droplet.

The SEM images of the cut capsule revealed that most of the PTFE particles were embedded inside the wax layer (Supplementary Fig. 6a). In other words, the particles were not visible on the outer surface of the encapsulation. Additionally, particles were not visible to protrude on the inner surface of the capsule wall (Supplementary Fig. 6b). This further indicates the almost complete submergence of PTFE particles within the wax. A similar experiment was performed with Glaco-coated glass beads. We found that a very small portion of particles (< 10% of the size) is exposed to the air, indicating flow is taking place primarily through the particle layer with some flow over the particles. The particle is observed to protrude at both the inner and outer surface of the capsule (Supplementary Fig. 6c). The different submergence behavior between the PTFE and glass beads is owing to the differences in their shape and wettability. The PTFE particles are mostly flat with irregular shapes. In contrast, glass beads are spherical. The water contact angle on PTFE is 120°, whereas, on the Glaco-coated glass surface, it is 150°.

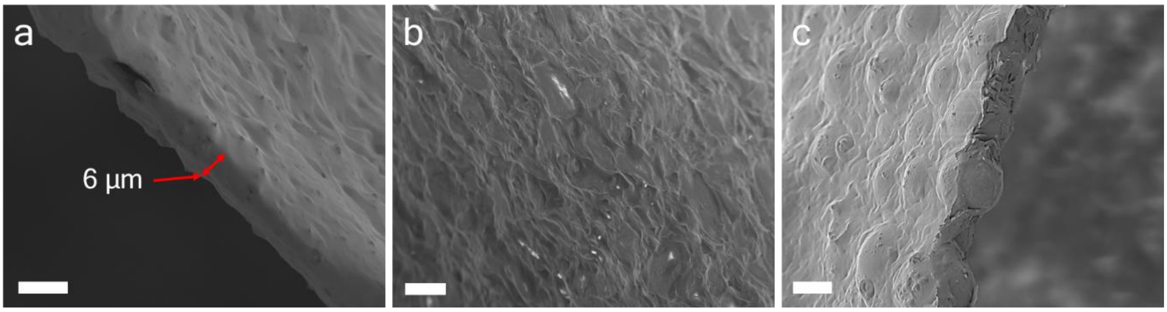

Supplementary Fig. 6: SEM of wax shell. a, Side view of cut wax capsule for 800 nm particle size. Scale bar = 10 µm. **b,** The inner side of the wax capsule where no PTFE particle is observed. Scale bar = 20 µm. **c,** Monodispersity of Glass beads on droplet surface for wax capsule LMOI. Scale bar = 40 µm.

Particle density in LMOI

The particle density of LMOI will be slightly different from the respective LM. This is because the LM settles down and contact angle (θ_{LMOI}) decreases during the oil rise. The surface area of the LMOI increases and thus interparticle distance also changes. The particle surface fraction

reduces with the LMOI formation. The surface fraction of LMOI (ϕ_{LMOI}) can be approximated by $\phi_{\mathrm{LMOI}} = \phi_{\mathrm{LM}} A_{\mathrm{LM}}\ (A_{\mathrm{LMOI}})^{-1}$ where A_{LM} and A_{LMOI} are the surface area of LM and LMOI, respectively. Here, $A_{\mathrm{LM}} = \left(6\sqrt{\pi} V_{\mathrm{LM}}\right)^{\frac{2}{3}}$ where V_{LM} is the volume of liquid marble and A_{LMOI} is given by

$$A_{\mathrm{LMOI}} = (6(1 - \cos\theta_{\mathrm{LMOI}}) + \theta_{\mathrm{LMOI}}) \left(\frac{\pi}{3}\right)^{\frac{1}{3}} \left[\frac{V_{\mathrm{LMOI}}}{(1 - \cos\theta_{\mathrm{LMOI}})^2 (2 + \cos\theta_{\mathrm{LMOI}})}\right]^{\frac{2}{3}}$$

Thus, the particle surface fraction changes with the LMOI formation. However, the actual surface fraction may vary as there can be a multilayer coating of particles over an LM, which can give rise to a higher solid fraction LMOI.

Stability of LMOI

The density (i.e., the distance between nearby particles) affects the capsule thickness. Supplementary Fig. 5b shows the thickness of wax capsules with the change in mass loading (ML) for 35 µm PTFE particles. With the increase in mass loading, the interparticle distance decreases, and the thickness of the coating increases. We also found that the LMOI was formed up to a minimum mass loading of 3.12 µg mm^{-2}. Below this limit, we observed that cracks appeared promptly upon LM touching the oil-infused surface, indicating that the stability of the LMOI is directly related to the mass loading of the particles. Above this critical mass loading, all LMOI reported in the paper are mechanically stable.

Dispersity

The particles used in our study agglomerate below 1 µm in size, which was also reflected in the thickness data shown in Manuscript Fig. 1h. To investigate this further, we prepared wax capsules using 35 µm PTFE particles (non-spherical with a range of 25 µm to 45 µm) and examined them using SEM. The resulting image, presented in Supplementary Fig. 7a, showed

a thickness of around 25 μm, which is less than the average particle size, indicating the absence of agglomeration. However, when we prepared wax capsules using 800 nm particles, the resulting thickness was around 6 μm, strongly indicating the presence of agglomeration (Supplementary Fig. 6a). This finding was further supported when we collected 800 nm particles from the water interface onto a glass substrate. This was done using the Langmuir-Blodgett method (Supplementary Fig. 7b). A layer of 800 nm particles was formed on a liquid bath. Subsequently, the particle film was lifted from the bath on a glass substrate. SEM image of the lifted particle film shows agglomeration (Supplementary Fig. 7c). In contrast, for larger particles, the capsule thickness was nearly equivalent to the particle size, indicating the formation of a monolayer over the droplet interface. To directly visualize the monolayer formed over the droplet interface, we used superhydrophobic glass beads coated with Glaco mirror coat spray. The wax capsule prepared by such superhydrophobic glass beads shows a monolayer of the particles at the droplet surface (Supplementary Fig. 6c).

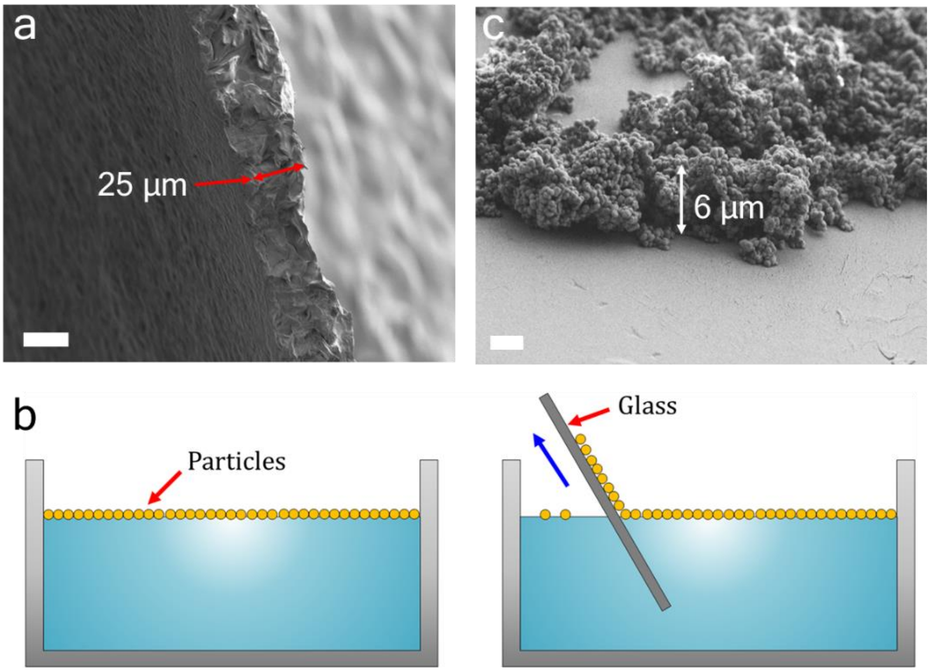

Supplementary Fig. 7: Agglomeration of particles. a, Thickness of the wax capsule for 35 μm particle size. Scale bar = 20 μm. **b,** Langmuir-Blodgett method, where particles were first stabilized on a water bath and then transferred onto a solid substrate by carefully dipping the

substrate into the liquid surface. **c,** Agglomeration size for 800 nm particles lifted on a glass slide using the Langmuir-Blodgett method. Scale bar = 2 μm.

Additionally, if the particles are polydisperse, it is expected to have higher stability than monodisperse particles. This can also be inferred from the data of critical pressure to break the capsule (Manuscript Fig. 6c). The capsule made by smaller particles (800 nm) gives nearly similar breakage pressure as 35 μm particles. This is due to the agglomeration of the particles for smaller sizes.

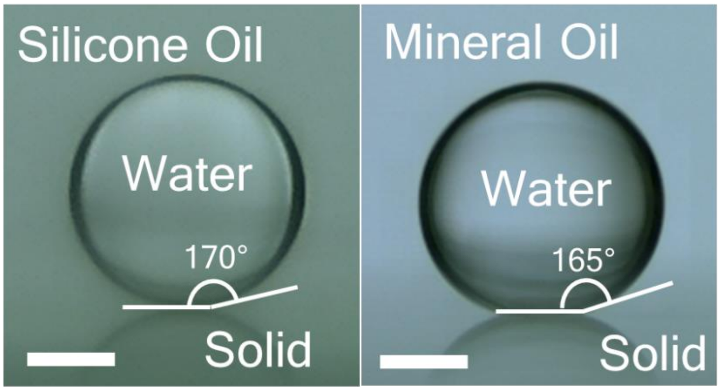

Supplementary Fig. 8: Contact angle at solid-liquid-liquid interface. The contact angle of the PTFE surface at the oil-water interface. Scale bar = 1 mm.

Supplementary note 4. The dynamics of cloaking

The cloaking process can be conceived as a two-step process. In such a process, a rapid covering of the exposed water-air surface by a thin layer of oil is expected within a few nm.[10] Subsequently, a thicker oil layer is formed by the flow of the oil through and over the particles. However, in our case, we don't observe a two-step process. All our experimental data show a single timescale for the cloaking process. To investigate our hypothesis further, we conducted an in-situ optical imaging analysis of the oil-cloaking process. Supplementary Fig. 9 (and Supplementary Movie 3) illustrates the cloaking process of 50 cP oil in Glaco-coated glass beads, which were stabilized on a flat water interface. The images clearly demonstrate that the oil layer predominantly passes through the gaps between the particles. However, it is worth noting that some flow over the particles can also occur, contingent upon the wettability of the particles.

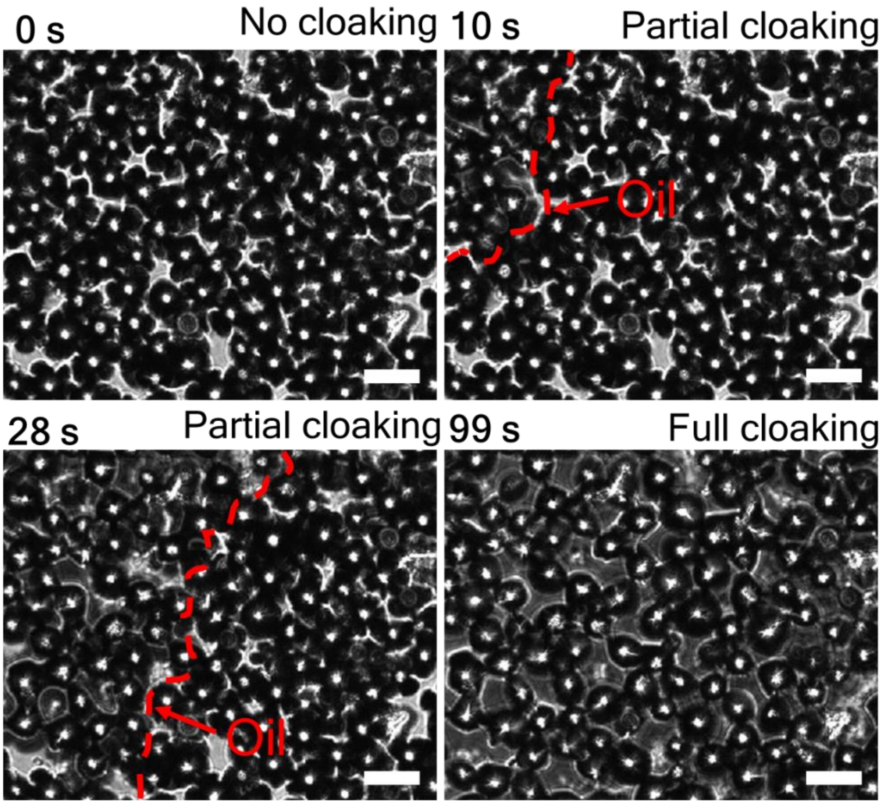

Supplementary Fig. 9: Microscopic movement of oil layer. The cloaking of 50 cP oil in a Glaco-coated glass particle-stabilized on a flat water interface. The dotted line represents the oil cloaked region. Scale bar = 100 µm.

Additionally, as the oil layer passes through the particles, the particle settlement happens. As represented in Supplementary Fig. 10, we tracked the movement of a single particle during the cloaking process. As the oil touches the particle, the particle changes its position due to sudden capillary force by the oil. Additionally, we observed the movement of particles even after oil completely passed through the particle layer. This after movement of particles may arise due to settlement of the particles at the minimum energy position.

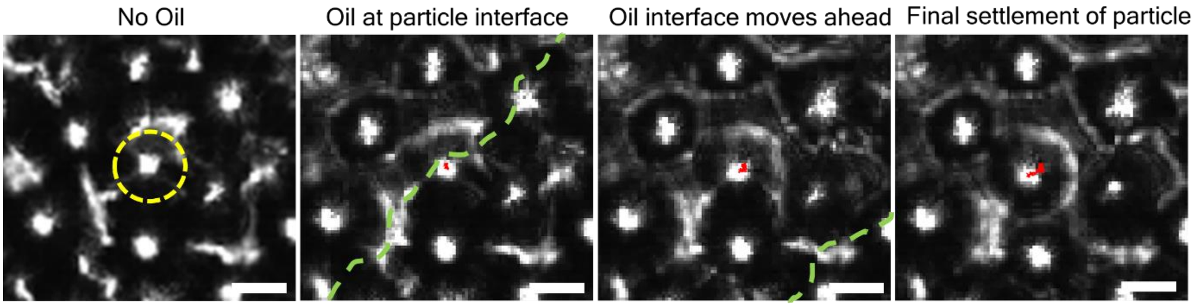

Supplementary Fig. 10: Particle settling due to oil cloaking. The particle tracking during oil movement. The dotted line represents the oil front and red line represents the total path particle took during the cloaking process. Scale bar = 50 µm.

To further validate the observed cloaking behavior, we performed a wax-based encapsulation of the particles and subsequently used scanning electron microscopy (SEM) to examine the results. The SEM images of the Glaco-coated glass beads provide compelling evidence of the complete encapsulation of the glass beads, except for their tops (Supplementary Fig. 11a, b), thereby confirming the substantial flow of oil through the particles. The SEM image of the PTFE particles reveals some minor protrusion of the PTFE particles through the wax layer, but we have not observed any significant contrast between the particle and wax layer that can conclusively prove about the nature of flow (Supplementary Fig. 11c). However, no flow over

the Glass beads top conclusively proves that the dominant mechanism of cloaking is flow through the particles and not over them.

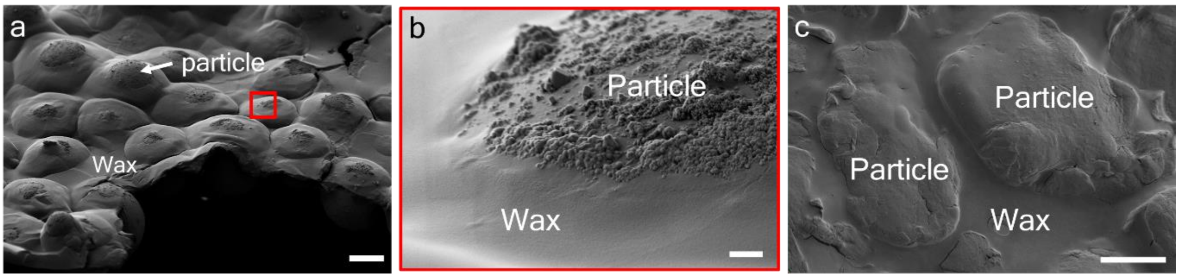

Supplementary Fig. 11: Coverage of the particle. a, The wax infusion in Glaco-coated glass beads where the top of the glass beads appeared to be free from wax. Scale bar = 20 µm. **b,** Zoomed in SEM image of the glass beads clearly shows no wax cover over the glass beads. Scale bar = 1 µm. **c,** SEM image of the PTFE particles shows protrusion of PTFE (non-spherical) through a wax layer. However, there may be wax flow over the particles. Scale bar = 20 µm.

Hence, we assume that most of the flow is primarily through the particles while very little flow is there over the particles. Based on the above observations, we have modeled the cloaking as a single-step process with the oil thickness approximately same as the particle (agglomerate) thickness, as shown in Supplementary Fig. 12.

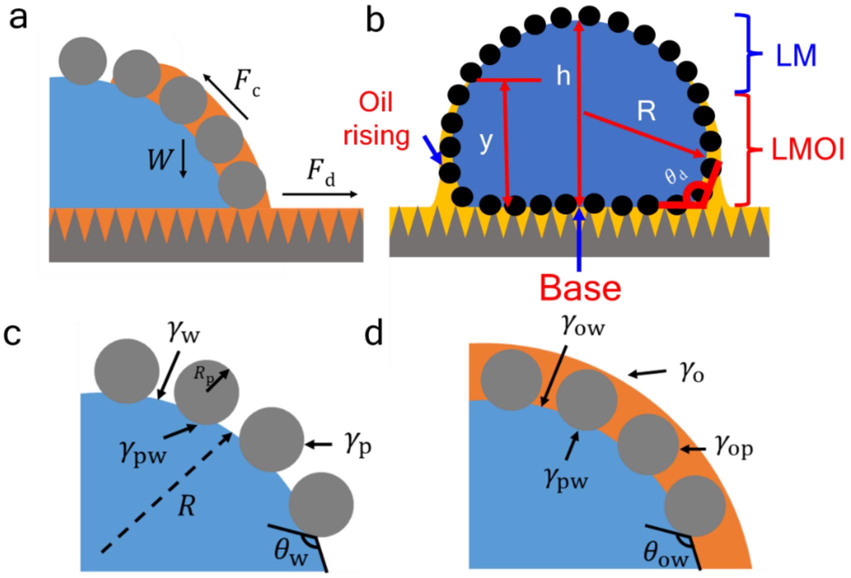

Supplementary Fig. 12: Surface energy contribution in LMOI. a, The effective forces during the oil rise. **b,** The schematic representation of dynamics of oil rise in LMOI. Schematic representation of different surface energy components of **c,** LM and **d,** LMOI.

The driving force for oil rise over the liquid marble is capillary force (F_c). Drag offered by the nanostructures in the oil-infused surface (F_d) and weight (W) of the oil are the resistive forces acting against the oil rise (Supplementary Fig. 12a). The drag induced by the LM particles is neglected as they are significantly larger than the nanostructures on the oil-infused surface. Thus, the equation of motion can be given as

$$F_\mathrm{c} - W = F_\mathrm{d} \tag{1}$$

For silicone oil of 10 cP viscosity and 35 μm particle size ($D_\mathrm{p} = 2R_\mathrm{p}$), the value of the Bond number ($Bo = \rho g D_\mathrm{p}^2 \gamma_\mathrm{o}^{-1}$) and the Galilei number ($Ga = \rho^2 g D_\mathrm{p}^3 \mu^{-2}$) are 6×10^{-4} and 4×10^{-3}, respectively. Thus, capillary and drag forces are much stronger than the weight of the oil; thus, Eq. (1) can be modified as $F_\mathrm{c} = F_\mathrm{d}$.

The effective surface energy of the LM and LMOI can be derived by considering individual contributions from water, particle, oil, and interactions between them. Considering n number of spherical solid particles with radius R_p covering the droplet with radius R. The schematic representation of process dynamics is given in Supplementary Fig. 12b. As represented in Supplementary Fig. 12b, the whole system can be divided into three major parts, the base, LMOI part (up to which the oil rise happened; height y), and LM part (where oil encapsulation has not happened yet; height $(h - y)$). During the oil rise, the LMOI settles, and the apparent contact angle with the surface changes. Therefore, we define a dynamic contact angle θ_d. If the volume of the LMOI is Ω then the effective radius (R) is given by

$$R = \left[\frac{3\Omega}{\pi(2 - \cos\theta_\mathrm{d} + \theta_\mathrm{d})}\right]^{\frac{1}{3}} \tag{2}$$

Thus, the surface area of each individual part, the base (S_base), LMOI (S_LMOI), LM (S_LM) and total surface area (S_tot) is given by

$$S_{\text{base}} = \pi R^2 \theta_{\text{d}} \tag{3}$$

$$S_{\text{LMOI}} = 2\pi R^2(1 - \cos\theta_{\text{d}}) - 2\pi R(h - y) \tag{4}$$

$$S_{\text{LM}} = 2\pi R(h - y) \tag{5}$$

$$S_{\text{tot}} = \pi R^2\{2(1 - \cos\theta_{\text{d}}) + \theta_{\text{d}}\} \tag{6}$$

We assume that the number of particles in any given configuration is proportional to its surface area. Thus, the number of particles is given by

$$n_{\text{base}} = n\frac{S_{\text{base}}}{S_{\text{tot}}} \tag{7}$$

$$n_{\text{LMOI}} = n\frac{S_{\text{LMOI}}}{S_{\text{tot}}} \tag{8}$$

$$n_{\text{LM}} = n\frac{S_{\text{LM}}}{S_{\text{tot}}} \tag{9}$$

Similarly, the surface fraction (ϕ_{s}) of individual parts is given by

$$\phi_{\text{s,LM}} = \frac{n\pi R_{\text{p}}^2\theta_{\text{w}}}{S_{\text{tot}}} \tag{10}$$

$$\phi_{\text{s,base}} = \phi_{\text{s,LMOI}} = \frac{n\pi R_{\text{p}}^2\theta_{\text{ow}}}{S_{\text{tot}}} \tag{11}$$

Here, the water interface has a contact angle of θ_{w} with the particles (Supplementary Fig. 12c), while the oil-water interface has a contact angle of θ_{ow} with the particles (Supplementary Fig. 12d).

As represented in Supplementary Fig. 12c, the surface energy contribution from the particles submerged in the water can be calculated as $2\pi R_{\text{p}}^2 n\gamma_{\text{p}}(1 + \cos\theta_{\text{w}})$, where γ_{p} is the surface energy of the particles. Similarly, surface energy contribution from the particles exposed to air is given by $2\pi R_{\text{p}}^2 n\gamma_{\text{pw}}(1 - \cos\theta_{\text{w}})$, where γ_{pw} is the surface energy of the particle-water interface. The total surface energy of the LM (E_{LM}) part is defined as

$$E_{\mathrm{LM}}(y) = S_{\mathrm{LM}}\gamma_{\mathrm{w}}\left(1 - \frac{n\pi R_{\mathrm{p}}^{2}\theta_{\mathrm{w}}}{S_{\mathrm{tot}}}\right) + 2\pi R_{\mathrm{p}}^{2}n_{\mathrm{LM}}(\gamma_{\mathrm{p}}(1 - cos\theta_{\mathrm{w}}) + \gamma_{\mathrm{pw}}(1 + cos\theta_{\mathrm{w}})) \tag{12}$$

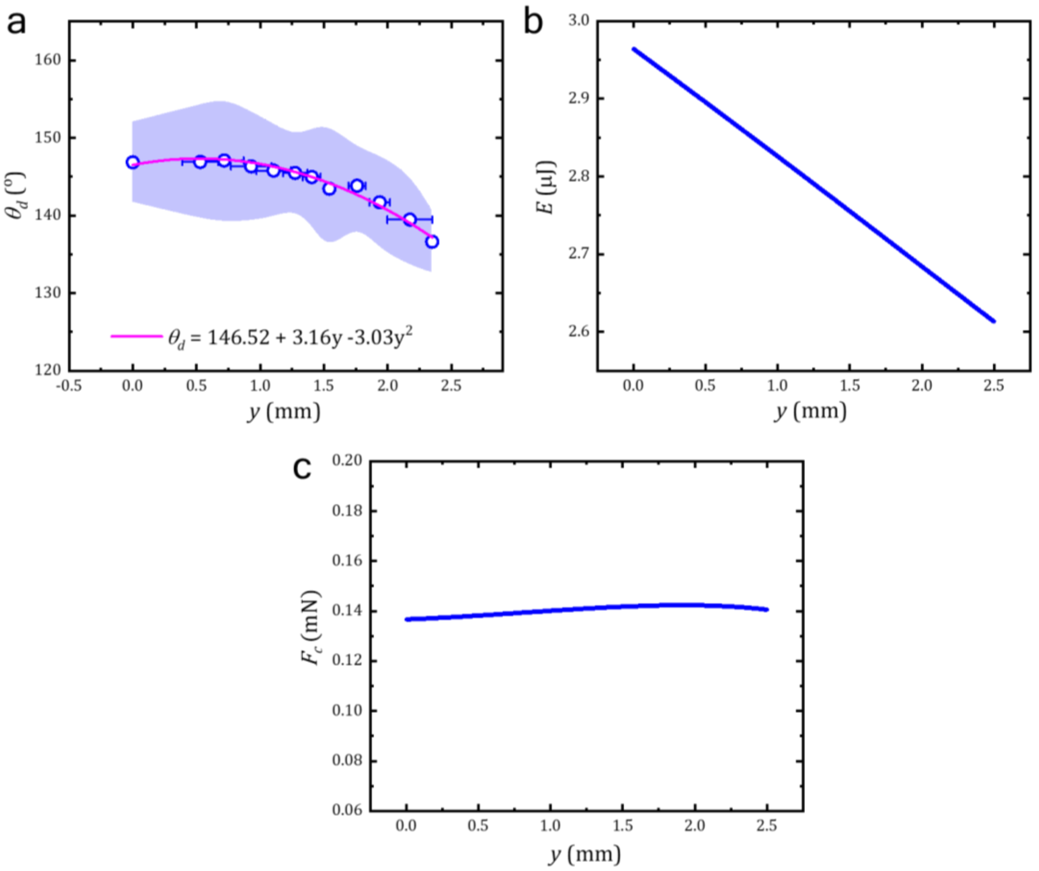

Supplementary Fig. 13: Capillary force during oil cloaking. a, The parabolic approximation of θ_d with the height variation. Measurements were carried out on n = 3 independent samples, and data are presented as mean values $\pm$ SD. **b,** The total energy and **c,** Capillary force variation with the height of LMOI. The overall variation in the capillary force with height is less than 4 %. The values of the different parameters used while calculating the above plots are $\Omega = 10$ µL, $h = 2.5$ mm, $\gamma_{\mathrm{p}} = 20$ mN m^{-1}, $\gamma_{\mathrm{o}} = 17.4$ mN m^{-1}, $\gamma_{\mathrm{w}} = 72$ mN m^{-1}, $\gamma_{\mathrm{ow}} = 41.97$ mN m^{-1}, $\gamma_{\mathrm{pw}} = 56$ mN m^{-1}, $\gamma_{\mathrm{po}} = 14.56$ mN m^{-1}, $\theta_{\mathrm{w}} = 120°$ *and* $\theta_{\mathrm{wo}} = 165°$.

Similarly, in the case of LMOI, the water-air surface energy is replaced by water-oil (γ_{ow}), the particle contact angle with the water-oil interface is θ_{ow}, particle-air surface energy is replaced by particle-oil surface energy (γ_{po}) and the additional component of oil surface energy (γ_{o}) is

added (Supplementary Fig. 12d). Thus, the effective total surface energy of the LMOI (E_{LMOI}) and the base part (E_{base}) is given by

$$E_{\text{base}}(y) = S_{\text{base}}\gamma_{\text{ow}}\left(1 - \frac{n\pi R_{\text{p}}^2\theta_{\text{ow}}}{S_{\text{tot}}}\right) + 2\pi R_{\text{p}}^2 n_{\text{base}}(\gamma_{\text{po}}(1 - \cos\theta_{\text{ow}}) + \gamma_{\text{pw}}(1 + \cos\theta_{\text{ow}})) \tag{13}$$

$$E_{\text{LMOI}}(y) = S_{\text{LMOI}}\gamma_{\text{o}} + S_{\text{LMOI}}\gamma_{\text{ow}}\left(1 - \frac{n\pi R_{\text{p}}^2\theta_{\text{ow}}}{S_{\text{tot}}}\right) + 2\pi R_{\text{p}}^2 n_{\text{LMOI}}(\gamma_{\text{po}}(1 - \cos\theta_{\text{ow}}) + \gamma_{pw}(1 + \cos\theta_{\text{ow}})) \tag{14}$$

Thus, the total energy variation with y is given by ($E(y)$)

$$E(y) = E_{\text{base}}(y) + E_{\text{LMOI}}(y) + E_{\text{LM}}(y) \tag{15}$$

The capillary force during the oil rise is given by $F_{\text{c}} = -\,dE(y)/dy$. In order to find the total energy, the variation of experimentally measured θ_{d} was plotted with the height variation, and then a parabolic fit was carried out (Supplementary Fig. 13a). For 10 μL volume and 35 μm particle size, the resulting $E(y)$ and F_{c} is represented in Supplementary Fig. 13b and Supplementary Fig. 13c, respectively. The variation of energy is nearly linear (Supplementary Fig. 13b); thus, the resulting capillary force is constant (Supplementary Fig. 13c).

Since the total energy varies linearly, the energy difference between the initial and final state (Δ_{LMOI}) drives the oil rise. The initial state is $E_{\text{i}} = E(0)$, and the final state is $E_{\text{f}} = E(h)$. Therefore, the driving capillary force can be given by

$$F_{\text{c}} = -\,\frac{dE}{dy} \approx \frac{\Delta_{\text{LMOI}}}{h} \approx \frac{E_{\text{i}} - E_{\text{f}}}{h} \tag{16}$$

Where h is the height of the droplet, y is the direction of oil rise and Δ_{LMOI} is the surface energy difference between two states.

The viscous drag force can be approximated as Poiseuille's law. The velocity gradient in the oil-infused surface is in the order of $\eta_o V p^{-1}$ where η_o, V, and p are the viscosity of oil, the velocity of oil, and the pitch between the nanostructures. The dissipation occurs over the area $\approx Dy$; thus, the viscous drag force is given by

$$F_d \approx \frac{\eta_o V D y}{p} \tag{17}$$

Balancing the capillary force with the viscous drag force results in

$$V \approx \frac{p\Delta_{\mathrm{LMOI}}}{\eta_o D h y} \tag{18}$$

The velocity of the oil rise is given by $V = y\,t^{-1}$ where t is the time instance of rise. Thus, the rise height (y) is given by

$$y \approx \left(\frac{p\Delta_{\mathrm{LMOI}}}{\eta_o D h} t\right)^{0.5} \tag{19}$$

Manuscript Fig. 2a, b represents the rising height of oil over an LM with time. The clear difference is present according to the viscosity of the oil utilized in making LMOI. Nondimensionalization of Eq. (19) reveals the time scale responsible for the oil rise in the LMOI system (τ_{LMOI}). Thus, normalizing the rise height (y) with the LMOI height (h) gives Eq. (20)

$$\frac{y}{h} \approx \left(\frac{t}{\tau_{\mathrm{LMOI}}}\right)^{0.5} \tag{20}$$

$$where, \quad \tau_{\mathrm{LMOI}} = \frac{\eta_o D h^3}{p\Delta_{\mathrm{LMOI}}}$$

As shown in Manuscript Fig. 2c, all data collapses into a single line when normalized rising height (y/h) is plotted against normalized time (t/τ_{LMOI}). Additionally, the slope of the data is approximately 0.5, which further validates Eq. (20).

With regards to the particle velocity, the careful observation of the particles at the air-oil-LM contact line suggests there is little to no change in particle position during the oil rise (Supplementary Fig. 10). This is due to very little gape and jamming of the interface with particles. The SEM image of the hydrophobic glass-bead-based capsule near the air-surface-LMOI contact line reveals a crowding of particles everywhere which restricts major movement of the particles (Supplementary Fig. 14).

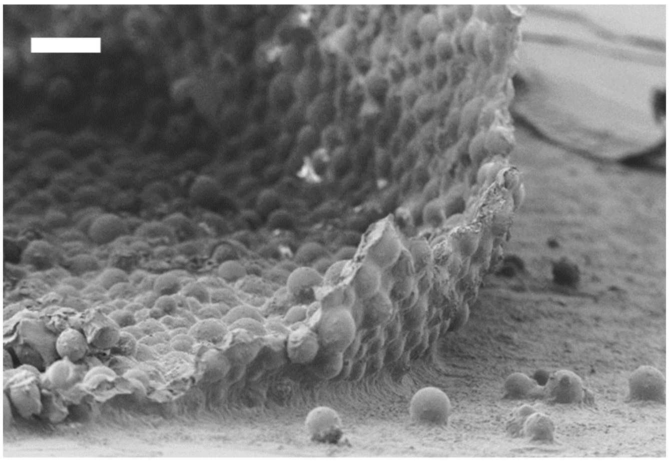

Supplementary Fig. 14: Crowding of the particles. SEM image of the capsule at the three-phase contact line where crowding of the particle is visible. Scale bar = 100 µm.

Supplementary note 5. Theoretical Model for Evaporation

The reduction in evaporation rate can be attributed to three effects. Firstly, the water vapor must pass through a layer of oil to evaporate. Secondly, the constriction of the diffusion path due to submerged particles. And third, the reduction in the effective surface area for evaporation because of the particle at the interface. However, as the particles are oleophilic, most of the particle is submerged in oil, while a tiny part touches the oil-water interface. Hence, only the first two effects play a major role in controlling the evaporation rate. As the droplet evaporates, it achieves a steady-state condition. Considering a symmetric profile, the continuity equation in spherical coordinates is given by Eq. (21), where N = molar flux of water vapor and r is the radius of the droplet.

$$\frac{1}{r^2}\frac{d}{dr}(r^2 N) = 0 \tag{21}$$

$$N = \frac{A_1}{r^2} \tag{22}$$

where, A_1 is the constant of integration. Taking the isothermal assumption, i.e., no change of diffusivity (D) and concentration (C) with temperature, Fick's equation can be represented by Eq. (23). Combining it with Eq. (22), gives Eq. (24).

$$\frac{dC}{dr} = -\frac{A_1}{D}\frac{1}{r^2} \tag{23}$$

$$N = -D\frac{dC}{dr} \tag{24}$$

Integration of Eq. (23) with the limit at $r = R$, $C = C_s$ and at $r = R + W$, $C = C_o$.

$$A_1 = D_O(C_s - C_o)\, R\left(1 + \frac{R}{W}\right) \tag{25}$$

where, D_O is the diffusivity of water in oil (m^2 s^{-1}), C_s is the saturation concentration of water in oil, C_o is the concentration of water vapor at the oil-air interface, and W is the width of the oil layer (without any particle constriction taking into account). Putting Eq. (25) in Eq. (22).

$$N = D_{\mathrm{O}}(C_{\mathrm{s}} - C_{\mathrm{o}})\,\frac{R}{r^2}\left(1 + \frac{R}{W}\right) \tag{26}$$

In order to get the net mass flow rate through oil, Eq. (26) can be integrated over the surface area (S) of the droplet.

$$\frac{dm_{\mathrm{O}}}{dt} = -\int^{S} M_{\mathrm{w}} N dS \tag{27}$$

where, m_{O} is the mass of water vapor passing through the water-oil interface and M_{w} is the molecular weight of the water vapor. Integrating Eq. (27) results in Eq. (28).

$$\frac{dm_{\mathrm{O}}}{dt} = -\,2\pi M_{\mathrm{w}} D_{\mathrm{O}}(C_{\mathrm{s}} - C_{\mathrm{o}})\,R\left(1 + \frac{R}{W}\right) \tag{28}$$

Similarly, the rate change of water evaporation through the oil-air interface can be obtained by integration of Eq. (29) with the limit at $r = R + W$, $C = C_{\mathrm{o}}$ and at $r \to \infty$, $C \to C_{\infty}$.

$$\frac{dm_{\mathrm{A}}}{dt} = -\,2\pi M_{\mathrm{w}} D_{\mathrm{A}}(C_{\mathrm{o}} - C_{\infty})\,(R + W) \tag{29}$$

where, m_{A} is the mass of water vapor passing through the oil-air interface, D_{A} is the diffusivity of water in the air ($m^2\ s^{-1}$) and C_{∞} is the concentration of water vapor in the atmosphere.

At a steady-state, rate of change of mass through the water-oil interface and oil-air interface should be equal. Thus,

$$\frac{dm_{\mathrm{A}}}{dt} = \frac{dm_{\mathrm{O}}}{dt} \tag{30}$$

$$C_{\mathrm{o}} = C_{\infty}\left[\frac{1 + \frac{R}{W}\frac{C_{\mathrm{s}}}{C_{\infty}}\frac{D_{\mathrm{O}}}{D_{\mathrm{A}}}}{1 + \frac{R}{W}\frac{D_{\mathrm{O}}}{D_{\mathrm{A}}}}\right] \tag{31}$$

As the system is at a steady state, the mass decrease can be determined from either Eq. (28) or Eq. (29). Till now, we have not considered the effect of particle constriction in oil, which may lead to a reduction in water vapor diffusion. Thus, we introduce the diffusion path correction factor (κ) in order to incorporate the reduction in diffusion area. The value of κ ranges from 0 (Full constriction) to 1 (no constriction). Putting the value of Eq. (31) in Eq. (29) and defining $m_\mathrm{A} = m$ with the introduction to κ

$$\frac{dm}{dt} = -\frac{2\pi M_\mathrm{w} D_\mathrm{O}(C_\mathrm{s} - C_\infty)}{\kappa} \frac{(R + W)}{\left(\frac{W}{R} + \frac{D_\mathrm{O}}{D_\mathrm{A}}\right)} \tag{32}$$

As $R = \beta V^{1/3}$ where β is the geometric parameter depending on the contact angle (θ) defined by Eq. (33).

$$\beta^3 = \frac{3}{\pi(1 - \cos\theta)^2(2 + \cos\theta)} \tag{33}$$

$$\frac{dm}{dt} = -\frac{2\pi M_\mathrm{w} D_\mathrm{O}(C_\mathrm{s} - C_\infty)}{\kappa} \frac{\left(\beta V^{1/3} + W\right)}{\left(\frac{W}{\beta V^{1/3}} + \frac{D_\mathrm{O}}{D_\mathrm{A}}\right)} \tag{34}$$

Eq. (34) represents the rate of change of droplet mass with time. This equation also remains valid for two extreme conditions, i.e., $W \to 0$ and $W \to \infty$.

Without any oil layer ($W \to 0$) and no constriction ($\kappa \to 1$), Eq. (34) is modified to Eq. (35), which is similar to what is reported in previous literature.[11,12] Here, C_s can be modified to the saturation concentration of water vapor at the water-air interface.

$$\frac{dm}{dt} = -2\pi M_\mathrm{w} D_\mathrm{A}(C_\mathrm{s} - C_\infty)\left(\beta V^{1/3}\right) \tag{35}$$

And with an infinite amount of oil ($W \to \infty$) and no constriction ($\kappa \to 1$), Eq. (34) is modified to Eq. (36). In which, the evaporation is entirely governed by solubility and diffusivity of water in oil.

$$\frac{dm}{dt} = -2\pi M_{\mathrm{w}} D_{\mathrm{O}} (C_{\mathrm{s}} - C_{\infty}) \left(\beta V^{1/3}\right) \tag{36}$$

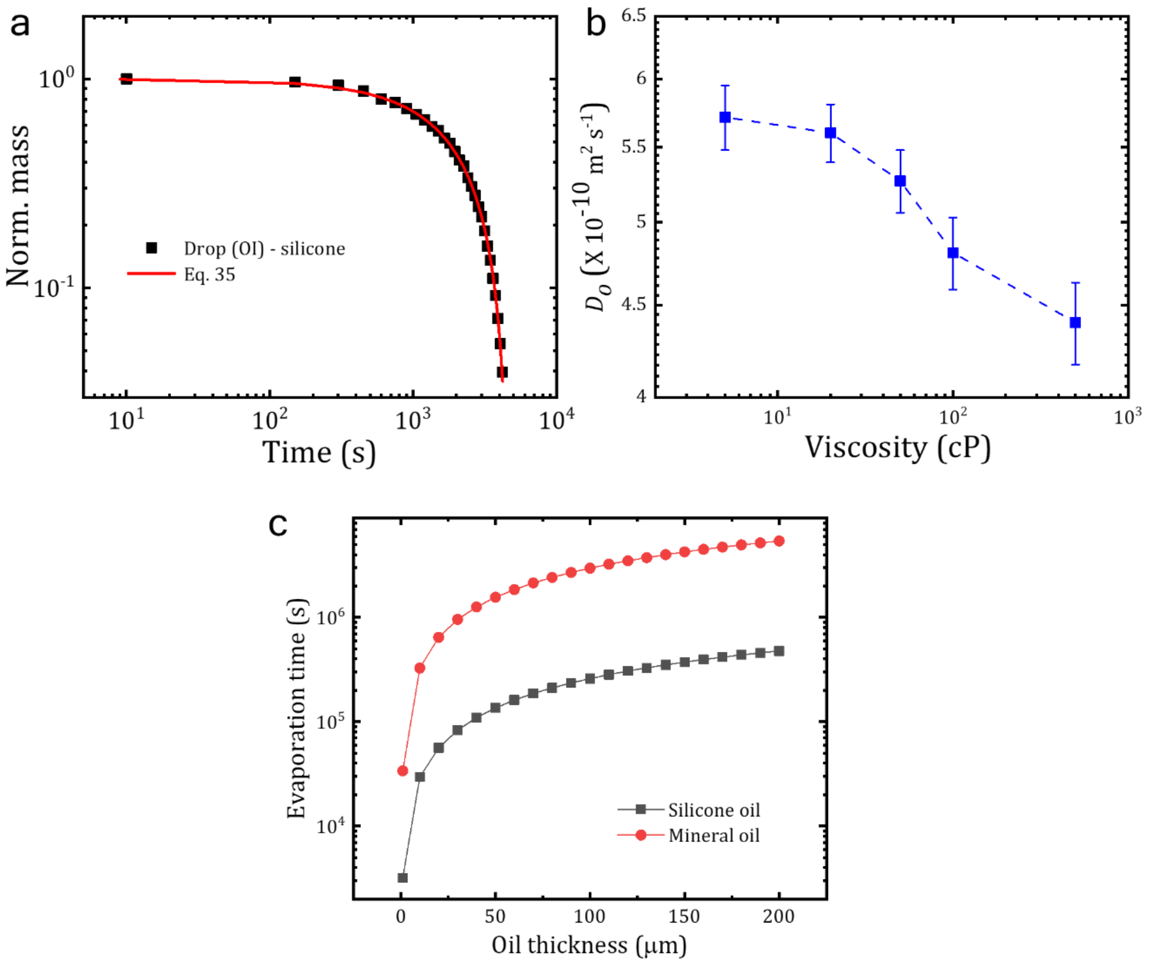

Supplementary Fig. 15: Effect of various parameters on evaporation. a, Comparison of the temporal evolution of normalized mass with a theoretical model for the bare water droplet on a silicone oil-infused surface. We hypothesized that coating is absent (or very thin) and found a value of water diffusion in the air (D_A) as ≈ 3×10^{-5} m^2 s^{-1} with R^2 = 0.9987. The value of D_A is approximately the same as reported in the literature.[13] Source data are provided as a Source Data file. **b,** The value of water diffusion coefficient in silicone oil (D_O) with various viscosity. Measurements were carried out on n = 3 independent samples, and data are presented as mean values ± SD. **c,** The variation of evaporation time with the change in oil thickness according to the theoretical model.

Supplementary note 6. Tuning the LMOI lifetime

There are several ways by which the droplet lifetime can be tuned.

(1) Changing the oil type (Manuscript Fig. 3a),

(2) Changing the viscosity of oil (Manuscript Fig. 3e),

(3) Changing the particle size (Supplementary Fig. 13c),

(4) Changing the particle mass loading (Manuscript Fig. 3f), and

(5) Decloaking on demand.

Supplementary Table 4 represents the typical tunability time scale for tunability according to this study.

**Method**	**Tunability**	**Remarks**
Oil type change	Days	Limited by diffusion and solubility of liquid in the oil
Oil viscosity	Min to hours	The viscosity of silicone oil is generally available with 10 cP gaps and thus controllable within several minutes to hours.
Particle size	Hours to Days	In the present study, particle size is controllable within 25 μm accuracy; thus, hours to days.
Mass Loading	Minutes	Mass loading of the LM can be tuned with a resolution of ≈ 2 μg mm^{-2}. Thus, the tunability is in minutes.
Decloaking of particles on-demand	Minutes	The time when decloaking happens decides the lifetime. Different evaporation rates before and after decloaking.

Supplementary Table 4: Tunable evaporation. Tunability of LMOI lifetime according to methods applied.

Supplementary note 7. Comparison of Evaporation Tunability

Supplementary Table 5 enlists droplet evaporation reported in various literature. The sessile droplet placed over an oil-infused surface shows nearly the same lifetime as the bare droplet. Additionally, LMOI's other counterparts, such as liquid marble (LM) and composite liquid marble, also suffer from rapid evaporation. The only technique that outperforms LMOI in reducing evaporation is submerging the droplet inside an oil bath. However, in the submerged case the tunability of evaporation is entirely absent in this technique.

Method	Material Used Droplet/Particles/Oil	Evaporation time	Droplet Volume Range	Temperature/Relative Humidity	Particle coating Thickness/ sizes	Remarks	Literature References
Sessile Drop coated with Oil	Polystyrene Colloidal solution/ – /Silicon Oil 100cst	≈500 s	0.1 μL	≈ 23°C / 47%	NA	Non-uniform coating. No tunability.	[14]
	Water/ –/K16256 Oil	≈500 s	0.087 μL	≈ 25°C / 50%	NA		[10]
	[1]Water/ – /Silicon Oil 350cst	≈84 min	4 μL	≈ 22°C / 38 %	NA		[4]
	[2]Water/ – /Silicon Oil 1000cst	160 min	10 μL	–	NA		[3]
	Water/silicone oil	10 h	10 μL	≈ 25°C / 95 %	NA		Experiments by authors

Liquid Marble (LM)	Water/PTFE/ –	26 – 60 min[!]	5 µL	≈ 26°C / 54 -60 %	20–100 µm/ 5–9 µm	NA	[15]
	Water/Graphene/–	5 – 50 min[!]	5 µL	≈ 23°C / 5 -87 %	– / 2–30 µm	NA	[16]
	PTFE/Agarose Hydrogel sphere	24 h	2 to 50 µL	≈ 37°C / NA	1 µm	Hydrogel sphere inside LM. No tunability	[17]
	PTFE	72 h	10 to 50 µL	≈ 37°C / NA	1 µm	Floating LM over the water bath. No tunability	[18]
Submerged LM	CD@POSS particles	≈ 1000 times normal LM	NA	≈ 26°C / NA	NA	No tunability<br><br>No Uniformity	[19]
Composite LM	PTFE/Mineral oil	≈ 3 h	10 µL	≈ 23°C / 50 %	35 µm	No tunability<br><br>No Uniformity	Experiments by authors
**LMOI**	**PTFE particles with silicone/mineral oil or wax**	**12 days (10 µL & 35 µm shell)**	**14 nL to 200 µL**	**≈ 25°C / 50 %**	**5 to 200 µm**	**Tunable, Uniform**	**This work**

*NA- not applicable;[!] – time reported before LM buckling;**[1] –** Oil coated on the hydrophobic surface;**[2] –** Oil coated (base coating ≈ 16µm) on the structured surface;

Supplementary Table 5: Comparison of evaporation rate by different techniques. The composite LM experiments was done by authors of this paper as the data of composite LM evaporation is not available in literature (marked as “experiments by authors” in table).

Supplementary note 8. Comparison with Other Crystal Growth Techniques

Supplementary Table 6 reviews various crystal-growing techniques. The commonly used bulk evaporation often suffers from the lack of evaporation control. Additionally, the desired size and uniformity of the crystal are also hard to achieve. Crystal growth and evaluating reaction kinetics need a controlled evaporation of the droplets. Control of evaporation is of utmost importance in single crystal growth as it ensures the optimum growth rate. Previously reported methods of crystal growth, such as submerging LM or droplets inside an immiscible phase, cannot tune the evaporation rate.[2] Such techniques provide an extremely low evaporation rate; thus, the time taken to form a single crystal is very long.[19] The evaporation rate tunability also helps in creating crystals with different properties.[20] Moreover, most of the conventional techniques produce multiple single crystals rather than one big crystal, which needs further processing to ensure a larger crystal formation.[21] Additionally, harvesting a crystal from oil is very difficult and requires special protocols and complex accessories.[22,23] It is worth noting that LM-based crystallization has been reported but is limited to interfacial crystallization and polycrystalline salt.[24–26] However, there has been no demonstration of single crystal growth inside LM or composite LM.

Method	Material Used	Volume	Advantages	Disadvantage	Literature References
Bulk Evaporation	ε-Hexanitrohexaazaisowurzitane	20 mL	Easy, scalable, and fast	Multiple crystal, Crystal density changes with evaporation rate, Difficult to control evaporation	20

	Lysozyme, Alcohol Dehydrogenase, Bovine Serum Albumin	3000 mL	Scalable, simple handling	Multiple crystals, Smaller in size	27
Submerged Droplet (Vapour Diffusion)	Lysozyme	5 µL	Reduced evaporation rate, Less contamination	Multiple crystals, Shock nucleation, No tunability of evaporation	28
	Thaumatin	≈ 4 µL	Crystallization time can be changed with oil, Batch process	Initial screening and optimization are time taking	29
	Lysozyme, Ferritin, Apoferritin, Glucose isomerase	20 nL to 2000 nl	Crystallization time can be shortened by changing the droplet volume	Multiple crystals, Require robotic systems, Initial screening, and optimization are time taking	30
	Alcohol Dehydrogenase	2 µL	Less contamination, resistant to small changes in temperature	Multiple crystals, Special protocol is needed for harvesting; not useful if the crystal has a solubility in oil	22
Liquid Marble	Sodium Chloride	10 µL	Promote Interfacial crystallization	Not a single crystal, Polycrystalline Salt	24–26
**LMOI**	**Copper sulfate, Rochelle salt, Sodium Nitrate, Lysozyme Protein**	**14 nL to 200 µL**	**Tunable evaporation, Single crystal formation, No merging, Optimal evaporation rate, easy screening, Tunable size**	**Not useful if the crystal has a solubility in oil, most suitable for water-based solvents.**	**This Work**

Supplementary Table 6: Crystal growth. Comparison of different crystal growth techniques.

Supplementary note 9. Crystal Growth in Oil-coated Droplets

Depending on the contact angle, the evaporation rate near the contact line is known to be different from the rest of the droplet surface. This is because vapor diffusion in air dominates the process. However, in our case, the evaporation rate is significantly lowered due to the oil layer. This is due to the very low saturation concentration of water in oil. This saturation-dominated slow evaporation rate significantly reduces (eliminates) the directional differences in the evaporation rate for the reported encapsulation scheme.

However, this will not be true for conventional oil covered compound droplets. We performed an experiment where a copper sulfate solution droplet was covered with silicone oil in a conventional compound droplet configuration. Here most of the oil is at the bottom near the contact line. The top of the droplet is covered with a very thin sub-micron thickness oil layer. Such a setup ensures minimal evaporation from the side while a higher evaporation rate from the top (Supplementary Fig. 16). This configuration resulted in faster evaporation, and single-crystal formation was not observed. We see polycrystalline fractal-like dendrite growth similar to water droplet freezing on oil-infused surfaces.[31] Thus, simple oil droplet-based encapsulation is not suitable for single crystal growth.

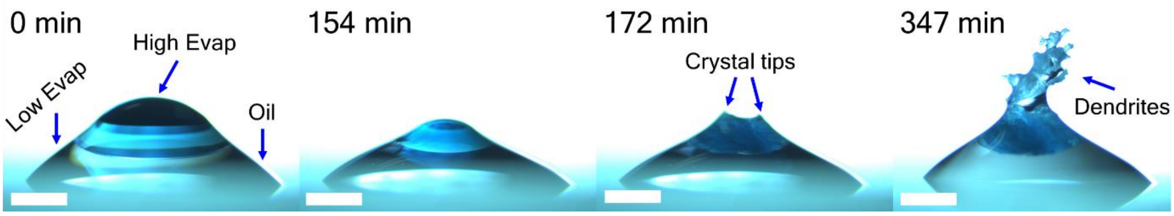

Supplementary Fig. 16: Morphology of crystal in oil-coated droplets without particles. Temporal evolution of the copper sulfate solution covered by silicone oil on a Teflon-coated glass surface. Scale bar = 1 mm.

**Single crystal**	**Concentration ($g\ ml^{-1}$)**	**Bare Droplet/LM/composite LM**	**Submerged Drop in Oil**	**LMOI (This work)**
Copper sulfate	0.3	No	Yes (> 2 months)	Yes (≈ 20 h)
Rochelle salt	1.5	No	Yes (> 2 months)	Yes (≈32 h)
Sodium Nitrate	0.9	No	Yes (≈ 13 days)	Yes (≈ 9 h)

Supplementary Table 7: Crystal growth time. Possibility of single crystal growth inside drops with various methods. The value in the bracket suggests the typical time frame for the single crystal growth.

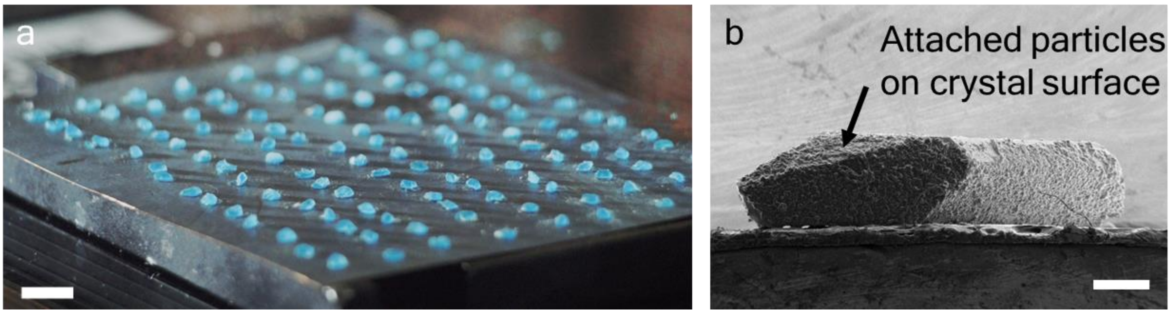

Supplementary Fig. 17: Crystals of copper sulfate. a, Array of a single crystal Copper Sulfate fabricated by automated setup. Scale bar = 1 cm. **b,** SEM image of Copper Sulfate produced in LMOI where, after complete evaporation, particles are seen to be attached on the crystal. Scale bar = 200 µm.

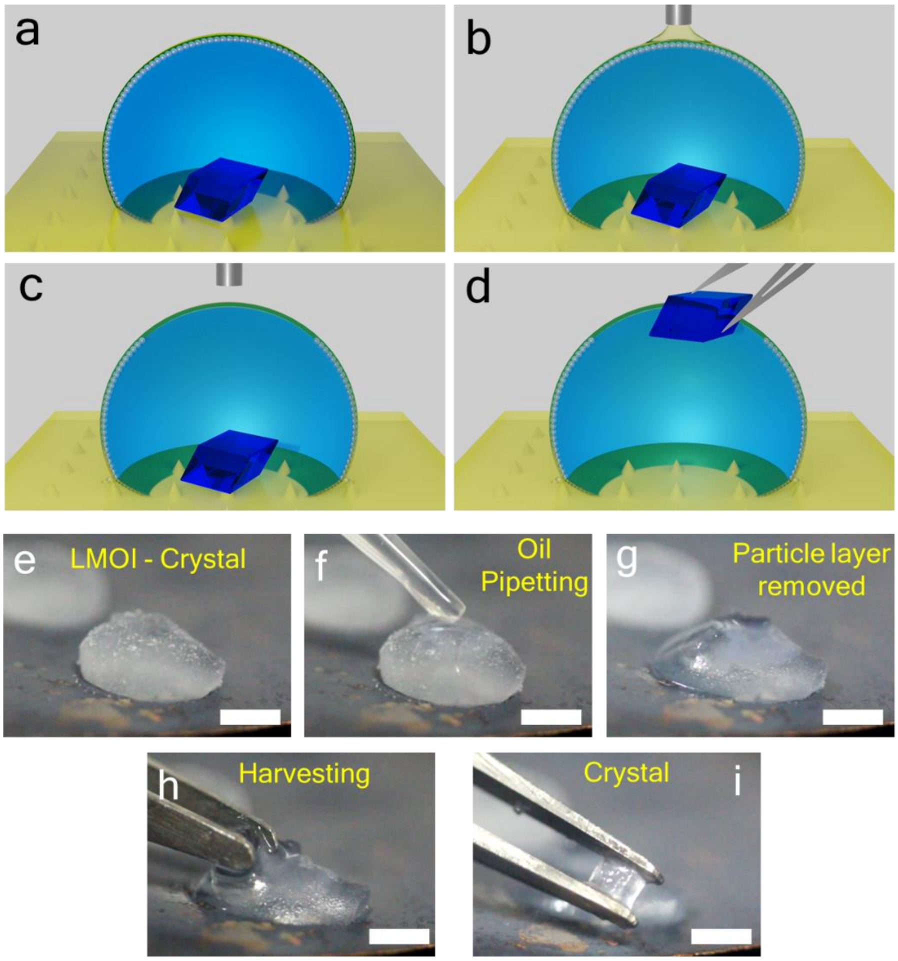

Supplementary Fig. 18: Crystal harvesting from LMOI. (a-d) Schematics representing the de-coating of particles and early harvesting of crystals. **a,** Formation of crystal occurs before total evaporation of LMOI. **b,** Extra oil is injected over a particle layer. **c,** An excessive amount of oil drags particles along with it and de-coats the LMOI. **d,** Crystal can be harvested. **(e-i)** Photographs of crystal harvesting. Scale bar = 1 mm.

Supplementary note 10. Comparison with other Bioreactor Platforms

Supplementary Table 8 reviews various platforms for bioreactors. Hanging droplet techniques have also been used in many biological applications, such as cell spheroid growth and protein crystallization.[32,33] These techniques require a humidity-controlled chamber. Thus, for long-term cell culture, special arrangements are required. Such as continuous injection or replacement of liquid to balance the evaporation rate.[33–37] This arrangement needs complex microfluidic devices, and the evaporation rate varies largely with the device designs.[33,34,36,37] The difficulty in handling and accessing droplets is another major concern in both hanging and submerged droplet techniques.[36,38] Similarly, the cell culture inside LM also suffers from a high evaporation rate. In order to reduce the evaporation rate, various techniques have been proposed, such as encapsulation of hydrogel[17] in the LM and stabilizing LM on the water bath.[18] However, such arrangements are limited to certain kinds of cell clusters,[17] and evaporation is still prevalent. Microfluidics-based cell cultures also suffer from evaporation and large osmolality shift for mammalian cell cultures.[39–41] The LMOI platform does not require any special cumbersome set up to mediate the evaporation rate. Additionally, LMOI possesses higher mechanical strength, which enhances the stability of bioreactors and prevents them from merging. However, on-demand merging is also demonstrated by using the stimuli-responsive oil. It is also possible to sample the liquid from LMOI without disrupting it.

Method	Material Used	Volume	Advantage	Disadvantage	Literature References
Hanging Droplets	A431.H9 cells	10 – 20 µL	Uniform cluster, High throughput	Require humidification chamber with media reservoir.	34

	HCT-116 eGFP cells	14 µL	Controllable, reproducible multi-spheroid culture	Require microfabrication and integrated microfluidics to compensate for evaporation	[33]
	NA	14 µL	Uniform cluster, multi-spheroidal culture (interconnected droplets)	Needs high humidity (> 95%) with constant feeding for evaporation control, Microfabrication	[37]
Liquid Marble	Olfactory ensheathing cells/PTFE particls	2 to 50 µL	Toroidal cluster formation	Require hydrogel inside LM to reduce evaporation. Evaporation is still prominent.	[17]
	Olfactory ensheathing cells/PTFE particles	10 to 50 µL	Uniform clusters	Floating LM over the water bath. Careful handling is needed. Evaporation is still prominent.	[18]
Microfluidics	Endothelial cell / PDMS	-	High cell volume to extracellular fluid volume	Large surface-to-volume ratio, promotes evaporation, sensitive to mammalian cells (due to osmolality shift in thin PDMS – even with high humidity)	[41,42]
	Mammalian cells / PDMS	-	High O_2 and CO_2 permeability	Chamber collapse due to evaporation, high osmolality shift, water has to pump through near cell culture to reduce osmolality shift	[39–41]
**LMOI**	**Ovarian Cancer cell & Yeast**	**14 nL to 200 µL**	**No need for Evaporation control, Prolonged incubation**	**Still need media exchange at several intervals**	**This work**

Supplementary Table 8: Cell growth techniques. Comparison with different cell growth techniques.

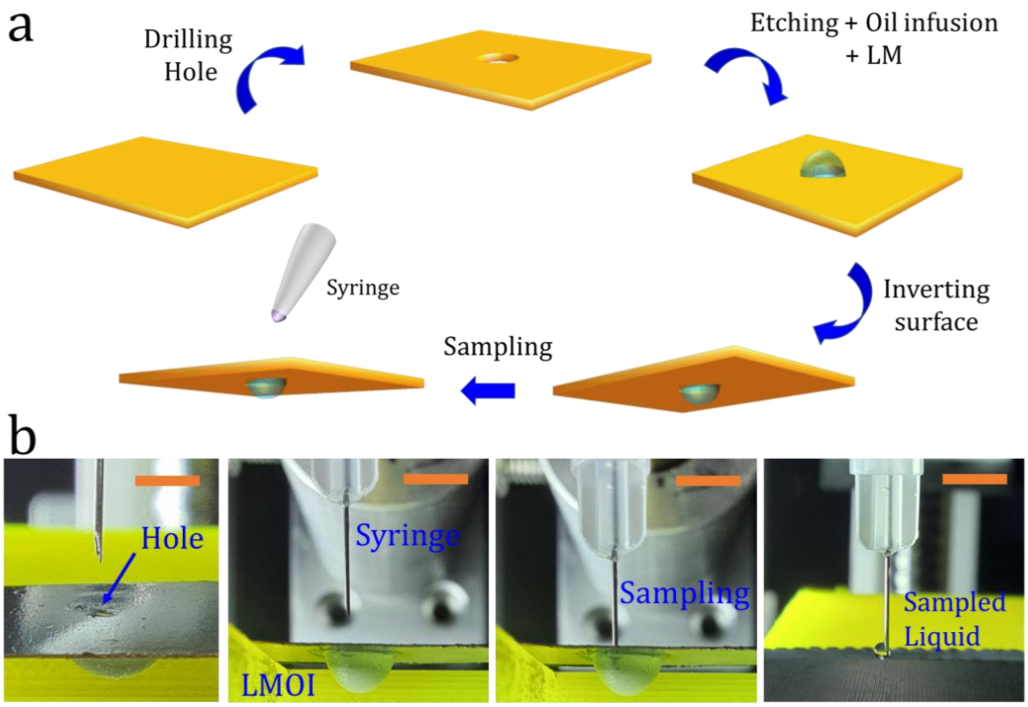

Supplementary Fig. 19: Accessing LMOI through surface engineering. a, Schematics representing the sampling process from hanging LMOI using drilled hole. **b,** A sampling of liquid from LMOI. The LMOI is stabilized on a surface with drilled hole. The syringe is inserted, and the desired volume of liquid can be sucked from LMOI. Here, the LMOI contains yeast solution. Scale bar = 3 mm.

Supplementary note 11. Comparison with Other Solid-Shell Techniques

Supplementary Table 9 reviews various solid shell-making techniques with the liquid core. However, most of the liquid/solid shell encapsulation techniques are either limited by the thickness of the shell or by the volume of the inner liquid. This is because these techniques cannot form tuneable and uniform shells over a wide range. Such tunability of solid and liquid shells over a wide range allows controlling processes within the encapsulated materials in a better manner. The unique ability of our method to tune and control the thickness (5 to 200 μm) of the encapsulation layer over a wide range of encapsulated volumes (4 orders of magnitude) enables previously unexplored applications such as single crystal growth.

Work/Literature	Material Used	Capsule size/Volume Range	Shell thickness	Remarks	Reference
Microfluidics	Aqueous poly(vinyl alcohol)	0.17 nL to 8.1 nL	7 to 50 μm	Permeable shell	[43]
	Aqueous poly(vinyl alcohol)	0.61 nL to 18 nL	1 to 67 μm	-	[44]
	Aqueous poly(vinyl alcohol)	113 nL	7 to 70 μm	Hollow shell	[45]
	Palm oil	38 nL	30 to 73 μm	Hermetic shell	[46]
	Aqueous poly(vinyl alcohol)	1.4 nL to 3.6 nL	9.7 to 28.4 μm	Permeable shell	[47]
	PNIPAM gel	0.52 pL to 0.52 μL	NA		[48]
Droplet Impact/jetting/needle	Alginate drop in calcium chloride solution	1.43 nL to 1.43 μL	140 μm to 1400 μm	Shell thickness depends on capsule volume	[49]

	Alginate drop in calcium chloride solution	24.4 µL to 44.6 µL	8 to 70 µm	-	[50]
	Glycerol – water mixture in silicone oil	6.21 µL to 13.43 µL	50 to 190 µm	-	[51]
	Paraffin Wax	10 µL	700 µm	Hermetic shell	[52]
	PLA + Silica	0.52 nL to 65 nL	≈ 10 µm	-	[53]
Stamping	Paraffin Wax	10 µL	500 to 1000 µm	Hermetic shell	[52]
3D printing	PLGA solution	14 nL	≈ 10 µm	-	[54]
**LMOI**	**PTFE particles with silicone/mineral oil or wax**	**14 nL to 200 µL**	**5 to 200 µm**	**Hermetic Shell**	**This work**

Supplementary Table 9: Capsule fabrication techniques. Comparison of different capsule-making techniques.

Supplementary note 12. The viscosity of wax with temperature

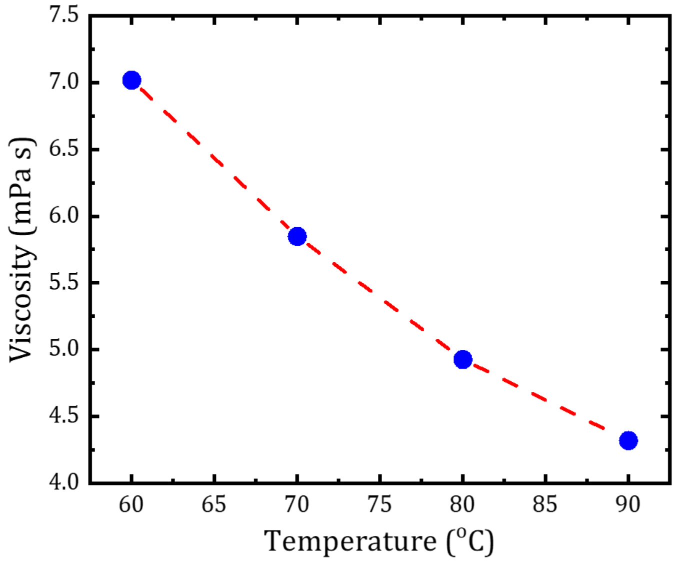

Supplementary Fig. 20: Wax viscosity. The variation of paraffin wax viscosity with temperature.

The melting temperature of the paraffin wax (Sigma-Aldrich) used in our experiments is 55 °C. The viscosity variation of the paraffin wax is represented in Supplementary Fig. 20. The viscosity measurement was carried out in an Anton Paar rheometer with a linearly changing shear rate from 1 s^{-1} to 100 s^{-1} in a cone plate configuration. Even close to the melting point (≈ 60 °C), we find a very low viscosity of wax (≈ 7.02 mPa s). At 70 °C, the viscosity of paraffin wax is around 5.84 mPa s. Thus, in order to ensure proper cloaking, the wax-infused surface was heated at 70 °C, and then LM was placed over it. At 70 °C, the cloaking happens within the span of 1.5 min, which is around 15 times less than the droplet evaporation time at the same temperature. Thus, the molten wax coats the LM before significant evaporation. The time scale for heat diffusion inside a droplet is given by $t_{\mathrm{diff}} \approx D^2 D_t^{-1}$ where D and D_{t} are the diameter of the droplet and the thermal diffusivity of the inner liquid. Taking the thermal diffusivity of water as 0.145 mm^2 s^{-1} with a droplet diameter of 2 mm, $t_{\mathrm{diff}} \approx 30$ s, which is of the same order of magnitude as wax cloaking time (≈ 90 s).[55] Thus, the droplet achieves an

equilibrium temperature before the complete cloaking. This ensures very little to no thermal gradient for the wax to freeze. Since the cloaking temperature (70 °C) is well above the melting point of wax (55 °C), the wax layer is expected to be in the molten state even with minor fluctuations in temperature. Additionally, we only observed the solidification when the whole setup was brought down to room temperature.

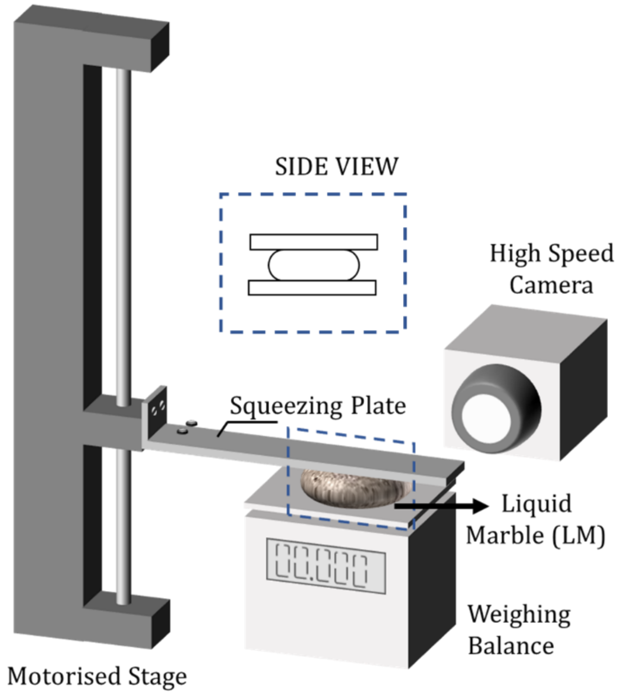

Supplementary Fig. 21: Critical pressure measurement. Schematic representing the critical pressure measurement setup.

Supplementary note 13. Mechanism of Release from Temperature-responsive Capsule

The release mechanism of the capsule depends on the melting of the wax shell. All the capsules were kept inside a water bath at the same time after reaching the bath temperature of 90 °C. The temporal evolution of the melting front is given by $L(t) \approx (\alpha_l t)^{0.5}$, where L, α_l, and t are the length of the melting front, thermal diffusivity in the liquid phase, and time instance, respectively.[56] Taking $\alpha_l \approx 0.19$ mm^2 s^{-1} [57] and thickness of capsule as $L \approx 200$ µm, the time for complete melting is in the order of 0.1 s, However, actual time is around 4 orders of magnitude higher ($\approx 10^3$ s) than the predicted by melting front equation. Thus, in our case, the mechanism of capsule disintegration is very different from melting-based disintegration. We believe that the disintegration is the consequence of thinning of capsule wall under the influence of Marangoni flows in the water bath (Supplementary Fig. 22). The shear of molten wax against the Marangoni flows results in thinning of the capsule and eventually disintegration. Since the shear thinning depends on the thickness of the shell wall, we observe an increase in disintegration time with increase in thickness. However, the exact modelling of the phenomenon is complex and out of scope for this paper.

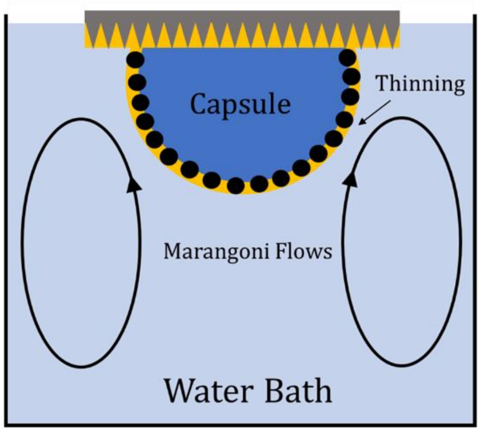

Supplementary Fig. 22: Capsule release mechanism. Schematics representation of capsule thinning through the Marangoni flow.

Supplementary note 14. **Cloaking on flat interface**

To ascertain the general applicability of our cloaking method, we conducted oil and wax infusion experiments on a flat interface. Initially, particles were stabilized on a water bath, and a wax-infused surface was then brought into contact with the particle bed. Upon heating the surface, the wax melted, and the infusion occurred inside the porous particle bed with the flat interface as well (Supplementary Fig. 23 and Supplementary Movie 3). Furthermore, we measured the average infusion velocity for the flat interface, which was found to be approximately ≈ 0.041 mm s^{-1}. This velocity is of the same order of magnitude as that observed in the case of low-viscosity oil (10 cP) using the LMOI setup (≈ 0.018 mm s^{-1}). The slight differences in infusion velocity are attributed to the distinct geometries and the differences in the relative surface areas of the interfaces involved in the experiments. The investigation into the generality of the cloaking method through oil infusion experiments on a flat interface provides compelling evidence that this approach is robust and adaptable across different interfaces, reinforcing its potential for practical applications.

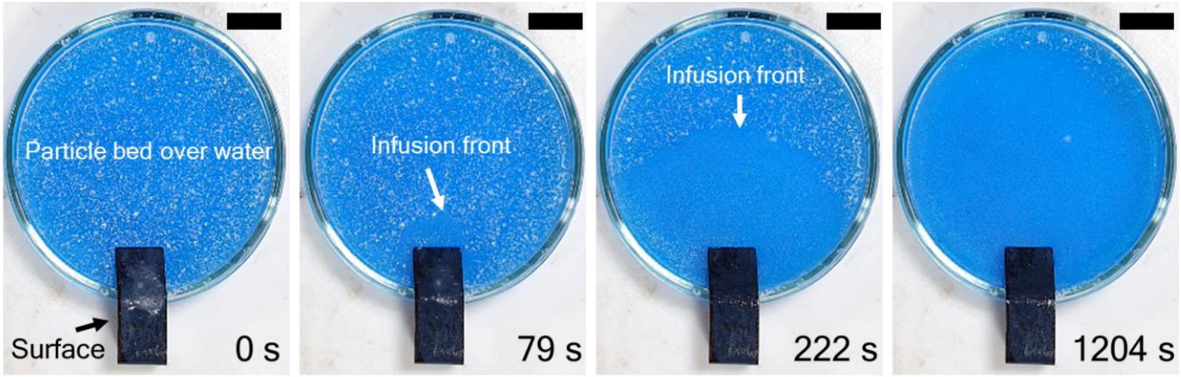

Supplementary Fig. 23: Flat interface cloaking. The temporal evolution of the wax infusion in a flat particle-coated water bath. Scale bar = 1 cm.

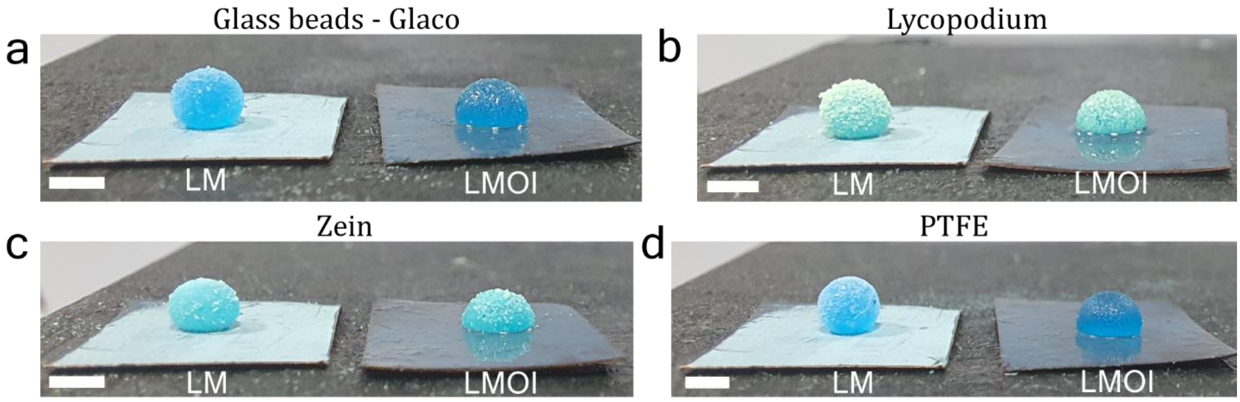

Supplementary Fig. 24: LMOI with different particles. The LM and LMOI for different hydrophobic particles such as **a,** Glaco coated glass beads, **b,** Lycopodium particles, **c,** Zein particles, and **d,** PTFE particles. Scale bar = 2 mm.

Supplementary references:

1. Roy, P. K. *et al.* Manufacture and properties of composite liquid marbles. *J. Colloid Interface Sci.* **575**, 35–41 (2020).
2. Bansal, S. & Sen, P. Axisymmetric and Nonaxisymmetric Oscillations of Sessile Compound Droplets in an Open Digital Microfluidic Platform. *Langmuir* **33**, 11047–11058 (2017).
3. Sahoo, S. & Mukherjee, R. Evaporative drying of a water droplet on liquid infused sticky surfaces. *Colloids Surfaces A Physicochem. Eng. Asp.* **657**, 130514 (2023).
4. Sharma, M., Mondal, S. S., Roy, P. K. & Khare, K. Evaporation dynamics of pure and binary mixture drops on dry and lubricant coated slippery surfaces. *J. Colloid Interface Sci.* **569**, 244–253 (2020).
5. Ge, Q. *et al.* Condensation of Satellite Droplets on Lubricant-Cloaked Droplets. *ACS Appl. Mater. Interfaces* **12**, 22246–22255 (2020).
6. Blanken, N., Saleem, M. S., Antonini, C. & Thoraval, M. J. Rebound of self-lubricating compound drops. *Sci. Adv.* **6**, (2020).
7. Blanken, N., Saleem, M. S., Thoraval, M. J. & Antonini, C. Impact of compound drops: a perspective. *Curr. Opin. Colloid Interface Sci.* **51**, 101389 (2021).
8. Liu, M. *et al.* Improvement of wall thickness uniformity of thick-walled polystyrene shells by density matching. *Chem. Eng. J.* **241**, 466–476 (2014).
9. Quéré, D. Inertial capillarity. *Europhys. Lett.* **39**, 533 (1997).
10. Günay, A. A., Sett, S., Ge, Q., Zhang, T. J. & Miljkovic, N. Cloaking Dynamics on Lubricant-Infused Surfaces. *Adv. Mater. Interfaces* **7**, 2000983 (2020).
11. Dash, S. & Garimella, S. V. Droplet evaporation dynamics on a superhydrophobic surface with negligible hysteresis. *Langmuir* **29**, 10785–10795 (2013).
12. Hatte, S., Pandey, K., Pandey, K., Chakraborty, S. & Basu, S. Universal evaporation dynamics of ordered arrays of sessile droplets. *J. Fluid Mech.* **866**, 61–81 (2019).
13. Armstrong, S., McHale, G., Ledesma-Aguilar, R. & Wells, G. G. Evaporation and electrowetting of sessile droplets on slippery liquid-like surfaces and slippery liquid-infused porous surfaces (SLIPS). *Langmuir* **36**, 11332–11340 (2020).
14. Gao, A. *et al.* Control of Droplet Evaporation on Oil-Coated Surfaces for the Synthesis of Asymmetric Supraparticles. *Langmuir* **35**, 14042–14048 (2019).
15. Tosun, A. & Erbil, H. Y. Evaporation rate of PTFE liquid marbles. *Appl. Surf. Sci.* **256**, 1278–1283 (2009).
16. Dandan, M. & Erbil, H. Y. Evaporation rate of graphite liquid marbles: Comparison with water droplets. *Langmuir* **25**, 8362–8367 (2009).
17. Vadivelu, R. K., Kamble, H., Munaz, A. & Nguyen, N. T. Liquid Marble as Bioreactor for Engineering Three-Dimensional Toroid Tissues. *Sci. Reports 2017 71* **7**, 1–14 (2017).

18. Vadivelu, R. K. *et al.* Generation of three-dimensional multiple spheroid model of olfactory ensheathing cells using floating liquid marbles. *Sci. Reports 2015 51* **5**, 1–12 (2015).

19. Zhao, Z. *et al.* Liquid Marbles in Liquid. *Small* **16**, 2002802 (2020).

20. Lee, M. H., Kim, J. H., Park, Y. C., Hwang, J. H. & Kim, W. S. Control of crystal density of ε-hexanitrohexaazaisowurzitane in evaporation crystallization. *Ind. Eng. Chem. Res.* **46**, 1500–1504 (2007).

21. Barros Groß, M. & Kind, M. Comparative Study on Seeded and Unseeded Bulk Evaporative Batch Crystallization of Tetragonal Lysozyme. *Cryst. Growth Des.* **17**, 3491–3501 (2017).

22. Chayen, N. E. The role of oil in macromolecular crystallization. *Structure* **5**, 1269–1274 (1997).

23. Douglas Instruments. https://www.douglas.co.uk/.

24. Roy, P. K., Shoval, S., Fujii, S. & Bormashenko, E. Interfacial crystallization in the polyhedral liquid marbles. *J. Colloid Interface Sci.* **630**, 685–694 (2023).

25. Bormashenko, E., Roy, P. K., Shoval, S. & Legchenkova, I. Interfacial crystallization within liquid marbles. *Condens. Matter* **5**, 1–11 (2020).

26. Roy, P. K., Legchenkova, I., Shoval, S. & Bormashenko, E. Interfacial Crystallization within Janus Saline Marbles. (2021).

27. Barros Groß, M. & Kind, M. From microscale phase screening to bulk evaporative crystallization of proteins. *J. Cryst. Growth* **498**, 160–169 (2018).

28. Blow, D. M., Chayen, N. E., Lloyd, L. F. & Saridakis, E. Control of nucleation of protein crystals. *Protein Sci.* **3**, 1638–1643 (1994).

29. D'Arcy, A., Elmore, C., Stihle, M. & Johnston, J. E. A novel approach to crystallising proteins under oil. *J. Cryst. Growth* **168**, 175–180 (1996).

30. Santarsiero, B. D. *et al.* An approach to rapid protein crystallization using nanodroplets. *J. Appl. Crystallogr.* **35**, 278–281 (2002).

31. Gandee, H. *et al.* Unique ice dendrite morphology on state-of-the-art oil-impregnated surfaces. *Proc. Natl. Acad. Sci. U. S. A.* **120**, e2214143120 (2023).

32. Ferreira, J., Sárkány, Z., Castro, F., Rocha, F. & Kuhn, S. Insulin crystallization: The route from hanging-drop vapour diffusion to controlled crystallization in droplet microfluidics. *J. Cryst. Growth* **582**, 126516 (2022).

33. Frey, O., Misun, P. M., Fluri, D. A., Hengstler, J. G. & Hierlemann, A. Reconfigurable microfluidic hanging drop network for multi-tissue interaction and analysis. *Nat. Commun. 2014 51* **5**, 1–11 (2014).

34. Tung, Y. C. *et al.* High-throughput 3D spheroid culture and drug testing using a 384 hanging drop array. *Analyst* **136**, 473–478 (2011).

35. Maayani, S., Martin, L. L. & Carmon, T. Water-walled microfluidics for high-optical finesse cavities. *Nat. Commun. 2016 71* **7**, 1–4 (2016).

36. Millet, L. J. & Gillette, M. U. Over a Century of Neuron Culture: From the Hanging

Drop toMicrofluidic Devices. *Yale J. Biol. Med.* **85**, 501 (2012).

37. Misun, P. M., Birchler, A. K., Lang, M., Hierlemann, A. & Frey, O. Fabrication and operation of microfluidic hanging-drop networks. *Methods Mol. Biol.* **1771**, 183–202 (2018).

38. Hong, J., Kim, Y. K., Won, D. J., Kim, J. & Lee, S. J. Three-dimensional digital microfluidic manipulation of droplets in oil medium. *Sci. Reports 2015 51* **5**, 1–11 (2015).

39. Forry, S. P. & Locascio, L. E. On-chip CO2 control for microfluidic cell culture. *Lab Chip* **11**, 4041–4046 (2011).

40. Thomas, P. C., Raghavan, S. R. & Forry, S. P. Regulating oxygen levels in a microfluidic device. *Anal. Chem.* **83**, 8821–8824 (2011).

41. Halldorsson, S., Lucumi, E., Gómez-Sjöberg, R. & Fleming, R. M. T. Advantages and challenges of microfluidic cell culture in polydimethylsiloxane devices. *Biosens. Bioelectron.* **63**, 218–231 (2015).

42. Yun, S. H. *et al.* Characterization and resolution of evaporation-mediated osmolality shifts that constrain microfluidic cell culture in poly(dimethylsiloxane) devices. *Anal. Chem.* **79**, 1126–1134 (2007).

43. Chen, P. W., Erb, R. M. & Studart, A. R. Designer polymer-based microcapsules made using microfluidics. *Langmuir* **28**, 144–152 (2012).

44. Xu, S. & Nisisako, T. Polymer Capsules with Tunable Shell Thickness Synthesized via Janus-to-core shell Transition of Biphasic Droplets Produced in a Microfluidic Flow-Focusing Device. *Sci. Reports 2020 101* **10**, 1–10 (2020).

45. Chen, R., Dong, P.-F., Xu, J.-H., Wang, Y.-D. & Luo, G.-S. Controllable microfluidic production of gas-in-oil-in-water emulsions for hollow microspheres with thin polymer shells. *Lab Chip* **12**, 3858–3860 (2012).

46. Ryu, S. A. *et al.* Biocompatible Wax-Based Microcapsules with Hermetic Sealing for Thermally Triggered Release of Actives. *ACS Appl. Mater. Interfaces* **13**, 36380–36387 (2021).

47. Chen, P. W., Brignoli, J. & Studart, A. R. Mechanics of thick-shell microcapsules made by microfluidics. *Polymer (Guildf).* **55**, 6837–6843 (2014).

48. Shah, R. K., Kim, J. W., Agresti, J. J., Weitz, D. A. & Chu, L. Y. Fabrication of monodisperse thermosensitive microgels and gel capsules in microfluidic devices. *Soft Matter* **4**, 2303–2309 (2008).

49. Martins, E., Poncelet, D., Marquis, M., Davy, J. & Renard, D. Monodisperse core-shell alginate (micro)-capsules with oil core generated from droplets millifluidic. *Food Hydrocoll.* **63**, 447–456 (2017).

50. Bremond, N., Santanach-Carreras, E., Chu, L. Y. & Bibette, J. Formation of liquid-core capsules having a thin hydrogel membrane : liquid pearls. *Soft Matter* **6**, 2484–2488 (2010).

51. Yin, S. *et al.* Triple-layered encapsulation through direct droplet impact. *J. Colloid Interface Sci.* **615**, 887–896 (2022).

52. Goertz, J. P., Demella, K. C., Thompson, B. R., White, I. M. & Raghavan, S. R. Responsive capsules that enable hermetic encapsulation of contents and their thermally triggered burst-release. *Mater. Horizons* **6**, 1238–1243 (2019).

53. Jiang, J. *et al.* High-Throughput Fabrication of Size-Controlled Pickering Emulsions, Colloidosomes, and Air-Coated Particles via Clog-Free Jetting of Suspensions. *Adv. Mater.* **35**, 2208894 (2023).

54. Gupta, M. K. *et al.* 3D Printed Programmable Release Capsules. *Nano Lett.* **15**, 5321–5329 (2015).

55. James, D. W. The thermal diffusivity of ice and water between -40 and + 60° C. *J. Mater. Sci.* **3**, 540–543 (1968).

56. Mansouri, L., Balistrou, M. & Baudoin, B. One-dimensional time-dependent modeling of conductive heat transfer during the melting of an initially subcooled semi-infinite PCM. (2017).

57. Murali, G., Mayilsamy, K. & Arjunan, T. V. An Experimental Study of PCM-Incorporated Thermosyphon Solar Water Heating System. *http://dx.doi.org/10.1080/15435075.2014.888663* **12**, 978–986 (2015).
