# Supplementary material for: Tunable encapsulation of sessile droplets with solid and liquid shells
Source: Nat Commun. 2023 Oct 13;14:6445. doi: 10.1038/s41467-023-41977-1 (PMC10575970; doi:10.1038/s41467-023-41977-1)
Supplement: Supplementary file 3 — Description of Additional Supplementary Information File [file 41467_2023_41977_MOESM3_ESM.pdf]

Description of Additional Supplementary Information

Title: Supplementary Movie 1:

Description: Failure and non-uniform oil layers in other methods such as oil wetting from the top and placing LM over an oil droplet.

Title: Supplementary Movie 2:

Description: LM + OI surfaces. The stable formation of LMOI with uniform coating. The video represents the oil rise in PTFE coated LM by 50 cP silicone oil infused surface.

Title: Supplementary Movie 3:

Description: The flow of 50 cP oil in Glaco-coated glass beads stabilized over a flat-water interface.

Title: Supplementary Movie 4:

Description: Automated setup for the scalable fabrication of LMOI for crystal making application.

Title: Supplementary Movie 5:

Description: Sampling from hanging LMOI for monitoring the media condition through the hole on OI surface.

Title: Supplementary Movie 6:

Description: The tunability of release from wax-based capsules. Two different thickness capsules filled with dye were placed in water at T ~ 90 °C. Encapsulated dye placed in water where lower thickness (~ 35 μm) capsule ruptures early, releasing the inner liquid. In comparison, a higher thickness (~ 200 μm) capsule ruptures after a prolonged duration.

Title: Supplementary Movie 7:

Description: On-demand manipulation and merging of stimuli-responsive LMOI based on ferrofluid.

Title: Supplementary Movie 8:

Description: The generality of the cloaking method. The present method works with the flat particle stabilized interface as well. The video represents the wax infusion in Glaco-coated glass beads stabilized water interface at 90 °C.
